# CXCR2 blockade overcomes the NETosis-mediated resistance to MEK inhibition in pancreatic cancer models

**DOI:** 10.1172/JCI196622

**Published:** 2026-03-19

**Authors:** Brian Herbst, Alex Blair, Yiming Li, Elizabeth M. Jaffee, Lei Zheng

**Affiliations:** 1 Department of Oncology and the Sidney Kimmel Comprehensive Cancer Center,; 2 Graduate Program in Cellular and Molecular Medicine,; 3 Pancreatic Cancer Precision Medicine Center of Excellence Program,; 4 Bloomberg-Kimmel Institute for Cancer Immunotherapy,; 5 Skip Viragh Center for Pancreatic Cancer, and; 6 Department of Surgery, Johns Hopkins University School of Medicine, Baltimore, Maryland, USA.; 7 Mays Cancer Center, University of Texas Health San Antonio MD Anderson, San Antonio, Texas, USA.; 8 Cancer Convergence Institute, Johns Hopkins University School of Medicine, Baltimore, Maryland, USA.

**Keywords:** Immunology, Oncology, Cancer, Cancer immunotherapy, Drug therapy

## Abstract

Single-agent anti-PD-1 antibodies are ineffective for pancreatic ductal adenocarcinoma (PDAC) due to the immunosuppressive tumor-microenvironment (TME). *KRAS* mutations contribute to the inflammatory TME and therapeutic resistance by upregulating IL-8 via MAPK pathways. Thus, this study attempted to overcome the resistance to anti-PD-1 antibodies by targeting downstream KRAS-effectors. The study found that the resistance to anti-PD-1 antibodies can be overcome through MEK1/2-inhibition. The combination of anti-PD-1 antibodies and MEK inhibitors displayed antitumor activity in Kras mutated (Kras^mut^) KPC mouse tumors, but not WT (Kras^WT^) Panc02 tumors. The combination of anti-PD-1 antibodies and MEK inhibitors induced recruitment of tumor-associated neutrophils (TANs) via CXCR2, an IL-8 receptor, and increased memory CD8^+^ T cells and IFN-γ production in treatment-sensitive tumors. However, larger tumors still resisted the combination of anti-PD-1 antibody and MEK inhibitor, likely due to hypoxia/necrosis-induced NETosis and associated paucity of CD8^+^ T cells. The subsequent addition of anti-CXCR2 antibody overcame this resistance by blocking TAN-infiltration to hypoxic/necrotic areas. Consistently, a risk-score based on the NETosis-MAPK signaling interaction is significantly associated with poorer survival in human PDAC. This study thus provides a new venue for overcoming resistance to strategies targeting KRAS signaling.

## Abstract 2

Anti-CXCR2 antibody overcomes the NETosis-mediated resistance to MEK inhibition treatment for the Kras-mutated PDAC by blocking TAN infiltration to hypoxic/necrotic areas.

## Introduction

Pancreatic ductal adenocarcinoma (PDAC) remains one of the most lethal solid-tumor malignancies. PDAC is projected to surpass breast, prostate, and colorectal cancer to become the second leading cause of cancer death in the United States by 2030 (1). Conventional therapies such as chemotherapy and radiation only marginally improve survival (2). Opportunities for small-molecule targeted therapies are limited by development of rapid resistance to single-agent inhibitors (3, 4). Most dauntingly, the recent breakthroughs with immune checkpoint blockade (ICB) therapies in other solid tumors targeting the PD-1/PD-L1 and CTLA-4 axes have failed in the vast majority of patients with PDAC (5, 6).

*KRAS* is a protooncogene that plays a key role in intracellular signal transduction. Greater than 90% of PDACs harbor mutant *KRAS* alleles, which lead to constitutive activation, driving cancer cell growth and survival (7). Although directly targeting mutant KRAS is promising, the small molecule inhibitors for the most common type of mutant KRAS in PDACs, *KRAS*^G12D^, or pan-KRAS mutant inhibitors are still at early stage of clinical development (3, 8). Past attempts have been made to develop small molecule inhibitors targeting the downstream pathways of mutant KRAS signaling, such as mitogen-activated protein kinase/extracellular signal-regulated kinase (MAPK/ERK) and PI3K isoforms (3, 9). Although these inhibitors have been shown to inhibit MAPK/ERK and PI3K isoforms mechanistically, they have produced limited responses in treating PDAC, despite success against other malignancies (9, 10).

The highly immunosuppressive nature of the tumor microenvironment (TME) is a primary mechanism for the resistance of PDAC to immune-based therapies (11, 12). During the initiation and progression of PDAC, oncogenic KRAS signaling fosters an immunosuppressive TME, characterized by desmoplasia, hypoxia, and pronounced myeloid cell infiltrate (13–15). PDAC is considered an immunologically ‘cold’ tumor, with low levels of effector T cells and low mutational burden. Secreted factors from PDAC cells attract and modulate myeloid cell populations with protumoral functions, including M2-polarized macrophages, both monocytic and PMN-myeloid-derived suppressor cells (MDSCs), and, notably, tumor-associated neutrophils (TANs). IL-8 and its murine homologs (CXCLs 1, 3, 5, and 7) bind to CXCR2 receptors found abundantly on granulocytes and serve as the main chemoattractants for circulating neutrophils and polymorphonuclear-MDSCs (PMN-MDSCs) to infiltrate the tumors (16–18). We previously reported that higher densities of TANs and increased levels of circulating IL-8 correlated with poorer survival in patients with PDAC who received ICB (19). Genetic deletion of CXCR2 in the genetically engineered *Kras*^G12D^ *p53 PDX1-Cre* (KPC) mice as well as pharmacological inhibition of CXCR2 in a pancreatic orthotopic mouse model with KPC tumors resulted in tumor suppression through enhanced infiltration and antitumor functions of CD8^+^ T cells (20, 21).

Neutrophils are the human body’s first defense for microbial infection and, more recently, were shown to have antitumor capabilities (22). Neutrophils kill tumor cells directly through generating ROS or their Ly6E^hi^ neutrophil subtype indirectly through coordinating the recruitment and function of other immune cells within the TME (23, 24). On the other hand, neutrophils are highly plastic cells. It has been shown that neutrophils have different functional statuses, including immunostimulatory (N1-like) and immunosuppressive (N2-like) subtypes (25). Nevertheless, how neutrophils are modulated by the oncogenic signaling in PDACs to alternatively exhibit immunosuppressive function remains unknown (19). More recently, the formation of neutrophil extracellular traps (NETs) in response to bacterial and fungal infection, so called NETosis, in the TME has been recognized. In addition to microbial moieties such as Lipopolysaccharide (LPS), NETosis can be induced by CXCR1/2, HMGB1, and oxidative free radical species (26, 27). Hypoxia and hypoxia-induced necrosis is a common feature of PDAC, and the subsequent release of HNBG1 and hypoxia-induced oxidative stress signals also may lead to NETosis (28, 29).

Therefore, in this study, we hypothesize that NETosis is the resistance mechanism used by PDACs against agents targeting both the KRAS downstream pathways, such as MEK inhibition, and immune checkpoints, such as PD-1 blockade, and that CXCR2 blockade may overcome this resistance mechanism.

## Results

### Targeted inhibition of oncogenic KRAS effectors identifies MEK1/2 as potent mediator of resistance to single-agent anti-PD-1 antibodies.

Since oncogenic KRAS employs a diverse set of downstream effectors and associated pathways to establish an immunosuppressive TME, before direct Kras inhibitors were available, we hypothesized that inhibition of various major Kras effectors in combination with anti-PD-1 antibody (aPD-1) would overcome the resistance to ICB treatment. To test this hypothesis, we first attempted to identify which effectors/pathways were responsible for resistance to aPD-1 blockade by systematic inhibition of Kras effectors and associated pathways. To minimize the effect of inhibitors of Kras effectors as single agents on tumor establishment, we chose to dose each inhibitor at or below a minimally effective dose, as determined through a literature review (9, 30, 31) (Supplemental Table 1; supplemental material available online with this article; https://doi.org/10.1172/JCI196622DS1). Furthermore, to maximize the translational efficiency of our study, we chose inhibitors that have already received full FDA approval or completed Phase III clinical trials in other tumor types whenever possible.

**Figure 1:**
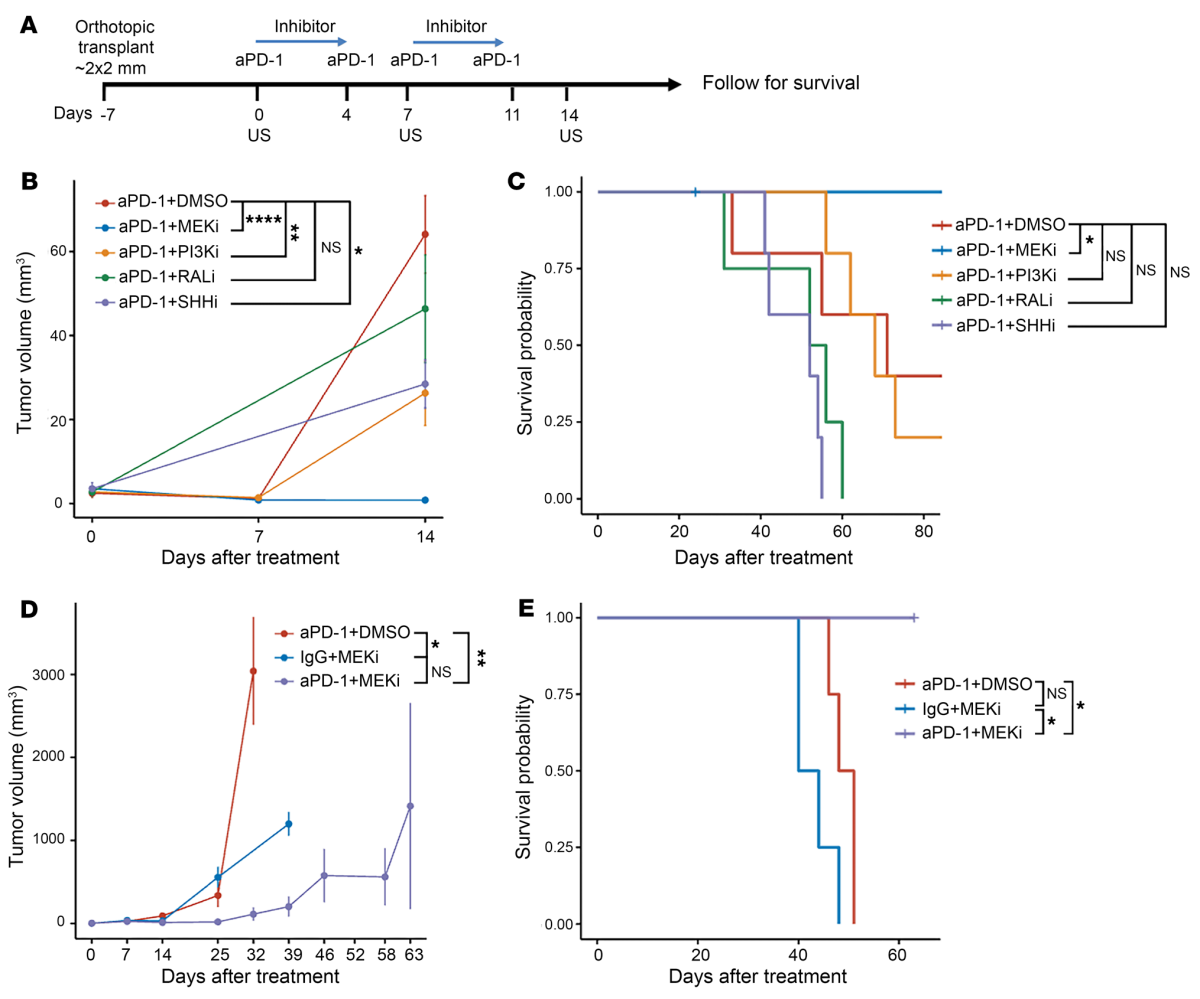
Inhibition of MEK1/2 signaling overcomes the resistance to anti-PD-1 blockade and, in combination with anti-PD-1 blockade, increases overall survival in the orthotopic KPC mouse model of PDAC. (**A**) Treatment schema of the orthotopic KPC mouse model with aPD-1 and KRAS effector inhibitors. The KPC001 cell line was used. Tumors were measured via ultrasound on days 0, 7, and 14 in **B** and weekly until Week 9 in **D**. (**B**) Representative tumor growth curves of aPD-1 combined with inhibition of KRAS effectors and associated pathways. *n* = 5 per group. (**C**) Kaplan-Meier survival curves of treatment groups in **B**. (**D**) Tumor growth curves of mice treated with aPD-1 ± MEKi in one representative experiment. *n* = 5 per group. Note that mice in the aPD-1 + DMSO group all met the survival endpoints, which were defined by morbidities instead of tumor size for the orthotopic model, and thus were euthanized by Day 32. (**E**) Kaplan-Meier survival curves of mice in **D**. Results are shown as mean ± SEM. Mixed effects model was used to compare tumor growth curves with Tukey’s corrections. For survival curves, Log-rank tests were performed followed by pairwise comparisons with Benjamin-Hochberg corrections. Key treatment groups were repeated at least twice. DMSO, vehicle; MEKi, MEK inhibitor; PI3Ki, PI3K inhibitor; RALi, RAL inhibitor; SHHi, Sonic hedgehog inhibitor; aPD-1, anti-PD-1 antibody; IgG, isotype control IgG for aPD-1. *P < 0.05; **P < 0.01; ***P < 0.001; ****P < 0.0001.

We have developed multiple tumor cell lines from the *Kras*^G12D^ *p53 PDX1-Cre* (KPC) mouse model that spontaneously develops invasive PDAC (32–34). Using an orthotopic model of murine PDAC derived from KPC cells (32) (Figure 1A), we assessed different inhibitors of KRAS downstream effectors. In this model, KPC cells are grown subcutaneously and then surgically transplanted into the pancreatic body of recipient mice. After allowing tumors to grow for 3 days, we treated mice with combinations of inhibitors and aPD-1 till Day 11, according to the schema in Figure 1A. Tumor growth was monitored via ultrasound, and antitumor activity was determined by tumor growth inhibition and subsequent survival analysis. In this screening experiment, our results showed that aPD-1 combined with the MEK1/2 inhibitor GSK211212/Trametinib (aPD-1 + MEKi), with PI3K inhibitor, or with sonic hedgehog (SHH) inhibitor, but not RAL inhibitor, delayed tumor growth compared with aPD-1 alone (Figure 1B) while aPD-1 + MEKi abolished tumor growth completely. Furthermore, only mice in the aPD-1 + MEKi group, both not in other combination groups, survived significantly longer compared with the aPD-1–alone group (Figure 1C). Therefore, we chose to validate the finding with aPD-1 + MEKi in the following experiments.

In additional KRAS downstream inhibitor screens using an aPD-1 isotype control given with the MEKi (IgG + MEKi), we found that MEKi alone delayed tumor growth not as much as the aPD-1 + MEKi combination (Figure 1D). Moreover, mice treated with IgG + MEKi developed macroscopic peritoneal metastases and died before Day 50, further reinforcing that MEKi alone is not responsible for the robust antitumor properties of the combination treatment. Nevertheless, we noticed that one mouse in the aPD-1 + MEKi group had tumor regrowth after Day 60, making the growth curves not statistically different between the aPD-1 + MEKi and IgG + MEKi groups, but overall survival still significantly different between these 2 groups (Figure 1, D and E).

### The combination of aPD-1 + MEKi displayed antitumor activities in the Kras-mutated KPC mouse tumor model but not the Kras WT Panc02 tumor.

**Figure 2:**
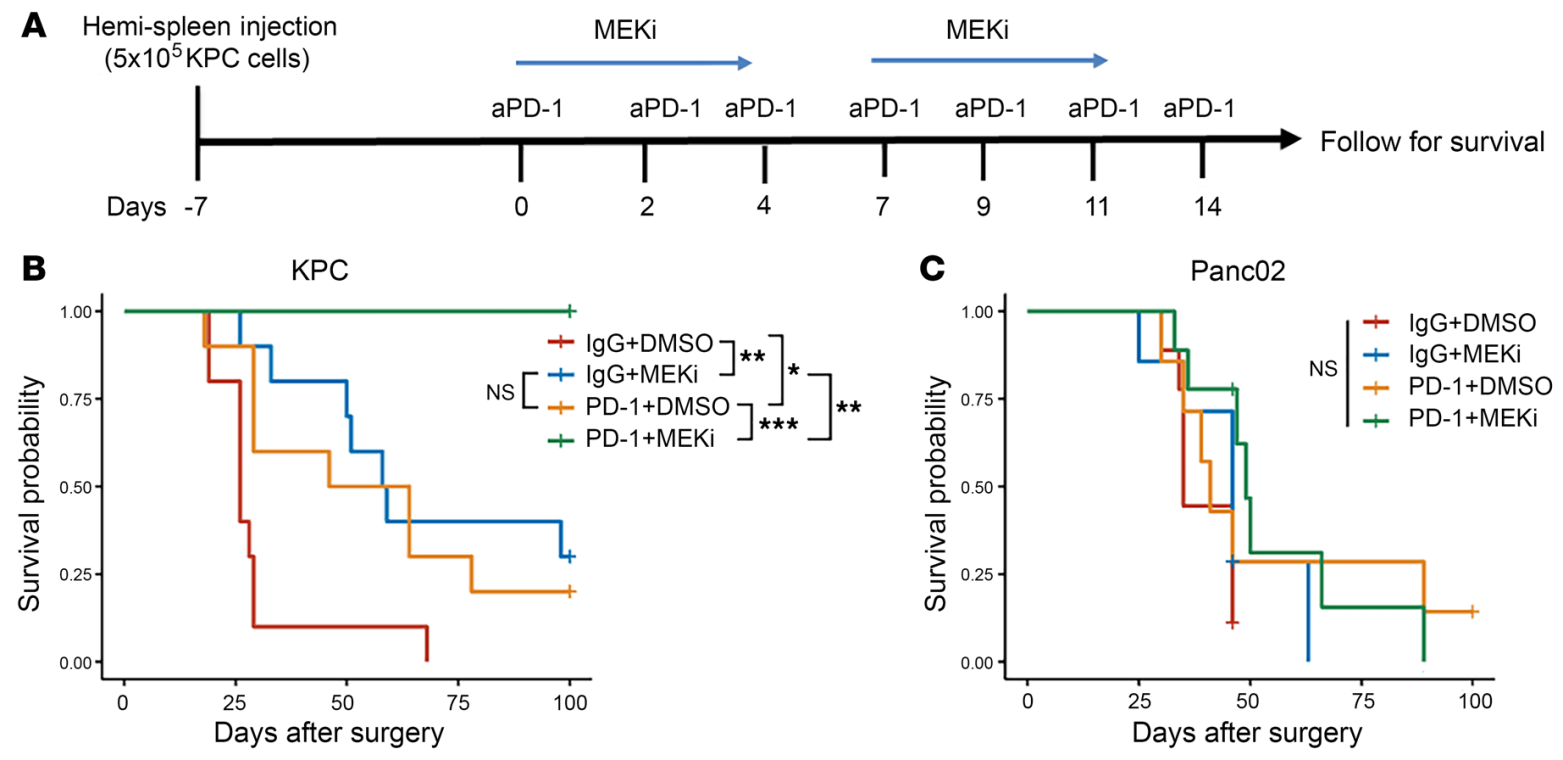
Combination treatment with anti-PD-1 antibody and MEK inhibitor prolongs survival in mice bearing KRAS^G12D^ PDCA liver metastases but not KRAS^WT^ PDAC liver metastases. (**A**) Treatment schema of the hemi-spleen liver metastasis model with aPD-1 and MEKi. For KPC tumors, 5 × 10^5^ KPC001 cells were injected per mouse; and for Panc02 tumors, 1.0 × 10^6^ cells were injected per mouse. Metastases were allowed to establish and grow for 7 days prior to treatment. Mice were followed for 100 days for survival endpoint. (**B**) Kaplan-Meier curves for KPC tumors. *N* = 10 mice/group. (**C**) Kaplan-Meier curves for Panc02 tumors. *N* = 10 mice/group. Log-rank tests were performed followed by pairwise comparisons with Benjamin-Hochberg corrections. *P < 0.05; **P < 0.01; ***P < 0.001.

As nearly 80% of patients with PDAC present with stage IV disease at the time of diagnosis, it is crucial for preclinical studies to include a demonstration of efficacy in a metastatic model of PDAC. Here, we employed a hemispleen model of diffuse liver metastasis (35), which reliably results from the injection of tumor cells in the splenic vasculature. After injection of tumor cells, metastases were allowed to establish and grow for 7 days before mice were randomized into control or treatment groups and dosed until Day 11 according to the schema in Figure 2A. We also used this as an opportunity to broadly test the hypothesis that MEK inhibition and aPD-1 blockade is dependent upon the dysregulated RAS-MEK-ERK signaling cascade driven by mutant KRAS. Therefore, mice were either injected with KPC tumor cells or the KRAS WT alternative Panc02 tumor cells.

Our results show that aPD-1 + MEKi is indeed efficacious in eliminating metastatic KPC liver metastases, as evidenced by 100% survival at 100 days after surgery and significant survival improvement compared with other treatment groups (Figure 2, B and C). In addition, treatment with aPD-1 or MEKi alone resulted in a modest survival benefit, albeit in a statistically significant manner, compared with IgG + DMSO control treatment, suggesting marginal benefit from single-agent treatment in the hemi-spleen model. In contrast, there were no significant differences in survival between treatment groups in the Panc02 liver metastasis model, thus supporting our hypothesis that the response to aPD-1 + MEKi is mutant KRAS-dependent.

### Identification of KPC cell lines in the orthotopic mouse models that are sensitive or resistant to the combination of anti-PD-1 antibody and MEK inhibitor.

**Figure 3:**
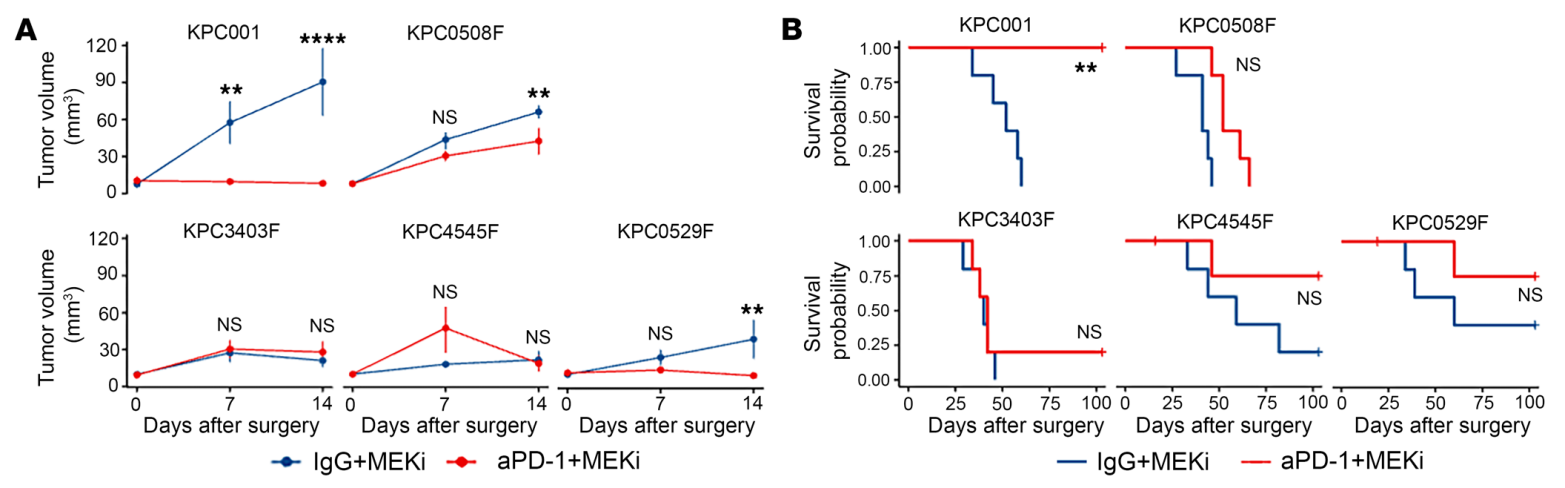
Identification of KPC cell lines in the orthotopic mouse models that are sensitive or resistant to the combination of anti-PD-1 antibody and MEK inhibitor. (**A**) Tumor growth curves for individual KPC cell lines in the orthotopic mouse model treated with aPD-1 + MEKi or IgG + MEKi. (**B**) Kaplan-Meier survival curves of mice in **A**. Experiments were done in duplicate. *n* = 5 mice/group. Growth curves were compared by the mixed effects model and survivals were compared by Log-rank tests. *P < 0.05; **P < 0.01; ****P < 0.0001.

Nevertheless, the phase 1 trial of the MEK inhibitor selumetinib in combination with aPD-1 pembrolizumab for advanced or metastatic solid tumors was stopped early because of insufficient efficacy (36). Prior to beginning mechanistic studies, we screened our library of KPC cell lines (34) with different metastasis potentials to identify control cell lines with an intrinsic resistance to the aPD-1 + MEKi combination treatment. The orthotopic models with these cell lines recapitulate TMEs in autochthonous models (37, 38). Using the same treatment schema as depicted in Figure 1A, we assessed 4 additional KPC cell lines with our orthotopic tumor model. Each group of mice received either aPD-1 + MEKi or IgG + MEKi to elucidate KPC lines that showed resistance to aPD-1 blockade in the context of MEK1/2 inhibition. Due to the technical complexity, we focused the remaining studies on KPC001BH (designated hereafter KPC_S) and KPC3403F (designated hereafter KPC_R) because they are the most clear-cut sensitive and resistant KPC cell lines, respectively. Note that there are statistically insignificant differences between aPD-1 + MEKi and IgG + MEKi treatment groups in both the tumor growth curves and survival data in the KPC-R tumor model (Figure 3, A and B) and that primary KPC_R tumors grow slowly, but with early metastases (34).

To assess if there was any difference in MEKi sensitivity between KPC_S (the original KPC cell line that is sensitive to aPD-1 + MEKi and KPC_R, we treated both of them with MEKi and compared pERK1/2 using an in-cell Western blot approach (Supplemental Figure 1). Interestingly, KPC_S showed a notable decrease in pERK1/2 signals even when treated with low concentrations of MEKi; whereas KPC_R showed a dose-dependent decrease in pERK1/2 signals. This result suggested that the pERK1/2 signaling in KPC_R is still altered by MEKi although with less sensitivity than KPC_S, and thus the resistance of KPC_R to the aPD-1 + MEKi combination was unlikely attributed to the effectiveness of targeted MEK inhibition alone.

### The combination of anti-PD-1 antibody and MEK inhibitor induces recruitment of TANs via CXCR2 in both sensitive and resistant KPC tumors while increasing CD8^+^ memory subtypes and IFN-γ production, specifically, in sensitive tumors.

**Figure 4:**
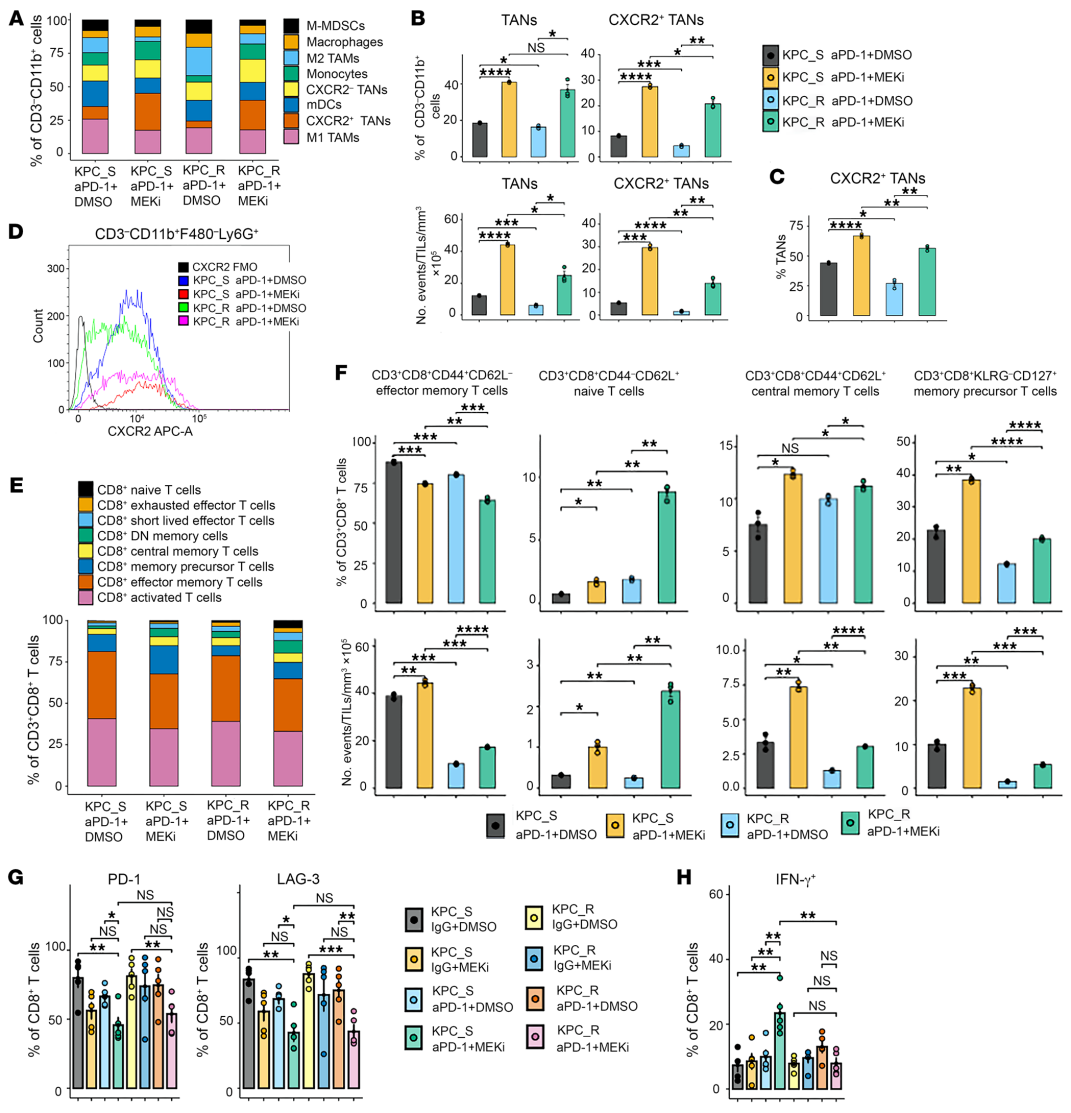
Immunophenotyping reveals the recruitment of TANs in both anti-PD-1 + MEKi–sensitive and –resistant tumors and an increase of memory T cells and IFN-γ production, specifically, in sensitive tumors. (**A**) Percentage compositions of CD11b^+^ myeloid cell subsets in each treatment group, as indicated. KPC_S: KPC001BH; KPC_R: KPC3403F, as indicated here and below. (**B**) Upper row: TANs and CXCR2^+^ TANs as percentages of myeloid cells. Bottom row: TANs and CXCR2^+^ TANs as density per tumor. (**C**) Percentage of CXCR2^+^ TANs among TANs. (**D**) Flow cytometry analysis of CXCR2 signal. *n* = 5 pooled tumors/group. (**E**) Mean percentage of CD8^+^ T cell subsets among CD8^+^ T cells. (**F**) Upper row, memory T cell subtypes as percentages of total CD8^+^ T cells. Lower row, memory T cell subtypes as cell density per tumor. (**G**) Percentages of PD-1^+^ and LAG3^+^ CD8^+^ cells among ex vivo stimulated CD8^+^ T cells in different treatment groups as indicated. (**H**) intracellular staining of IFN-γ in the same ex vivo stimulation experiment as G. Percentage of IFN-γ^+^ CD8^+^ T cells among CD8^+^ T cells were shown. Results are presented as mean ± SEM. *n* = 5 mice/group. Statistics were performed by Kruskal-Wallis test and multiple comparisons. *P < 0.05; **P < 0.01; ***P < 0.001; ****P < 0.0001.

To investigate the immune dynamics of microenvironment within tumors treated with aPD-1 + MEKi, we performed immunophenotyping of orthotopic tumors for both the myeloid and T cell compartments. We have previously demonstrated that the TME of the orthotopically implanted subcutaneous tumors is similar to that of the spontaneously formed KPC tumors (38). In addition, the pancreatic orthotopic model with a cubic of subcutaneously implanted tumor is resistant to aPD-1 therapy, whereas the subcutaneous implanted tumor model is moderately sensitive to aPD-1 therapy (39). We allowed the tumors to grow for 14 days prior to treatment to augment the size of tumor mass to enable tumor infiltrating immune cell analysis. MEKi treatment would be anticipated to decrease the myeloid cell infiltration shown to be induced by *Kras*^G12D^ in the KPC tumors. To the contrary, compared with aPD-1 + DMSO control tumors, neutrophils in the myeloid cell compartment in aPD-1 + MEKi–treated tumors increased significantly, both as a percentage among the CD11b^+^ myeloid compartment and as a measure of cell density per tumor weight (Figure 4, A and B). Specifically, such an increase in the neutrophil composition was attributed to an increase in the proportion of the IL-8 receptor CXCR2^+^ neutrophils, suggesting that aPD-1 + MEKi treatment induces preferential recruitment of CXCR2^+^ neutrophils to the tumor (Figure 4C). The median fluorescent intensity of CXCR2 suggested no difference in the expression of CXCR2 on TAN following the various treatments (Figure 4D). Consistently, we observed an increasing level of CXCR2, likely reflecting an increasing number of CXCR2^+^ TAN, and that of multiple CXCR2 ligands/mouse IL-8 orthologs, including CXCL1,3,5 according to the RT-PCR analysis of gene expression in the KPC tumors treated with MEKi (Supplemental Figure 1). As others have shown, the initial reduction in CXCR2 ligand expression upon MEKi treatment can be rapidly overcome by compensatory pathways (40).

Immunophenotyping of the T cell compartment focusing specifically on CD8^+^ T cells revealed that aPD-1 + MEKi treatment increased the proportion of CD44^+^CD62L^+^ central memory T cells and that of CD8^+^KLRG-CD127^+^ memory-precursor effector cells (MPECs), regardless of the sensitivity of KPC tumors to treatment (Figure 4, E and F). However, CD44^+^CD62L^–^ effector memory T cells were slightly decreased in aPD-1 + MEKi–treated tumors when viewed as a percentage of CD3^+^CD8^+^ T cells. In addition, CD3^+^CD8^+^ T cells (Supplemental Figure 2) as a whole were increased in aPD-1 + MEKi–treated tumors, which explained the slight decrease in the percentage of effector memory cells among CD8^+^ T cells. Supported by such a notion, the decreasing trend of effector memory T cells in aPD-1 + MEKi–treated tumors was reversed when normalized to cell density per tumor weight (Figure 4F). Most importantly, aPD-1 + MEKi treatment led to significantly higher percentage or density of all memory subtypes of CD8^+^ T cells, including central memory, effector memory, and memory precursor subtypes in KPC_S tumors compared with KPC_R tumors. These results suggested that aPD-1 + MEKi treatment induces memory T cell infiltration into the KPC tumors, particularly in those sensitive to treatment as opposed to those resistant to treatment. The underlying mechanism warrants further investigation. Failure to induce memory T cell infiltration may be a more direct reason for the treatment resistance in KPC_R.

To assay CD8^+^ T cell functional status, we employed the above-described hemispleen model of liver metastases, which give a larger yield of tumor-infiltrating CD8^+^ T cells than orthotopic tumors. After allowing tumors to grow for 10 days after injection, livers were harvested and processed individually for ex vivo stimulation with CD3/CD28 Dynabeads followed by intracellular staining for IFN-γ expression and small selection of inhibitory checkpoint markers. The effect of MEKi alone was similar in KPC_S and KPC_R tumors and resulted in reduced expression of both PD-1 and LAG-3. The combination aPD-1 + MEKi offered a further significant reduction of expression of both markers (Figure 4G). Interestingly, despite the reduction in PD-1 and LAG-3 expression in both KPC cohorts, the only CD8^+^ T cells to produce IFN-γ at a significant level were from aPD-1 + MEKi–sensitive tumors (Figure 4H), suggesting that a mechanism of inducing memory T cell infiltration, independent from the T cell exhaustion pathway, may underlie the antitumor immune response to aPD-1 + MEKi.

### Anti-CXCR2–blocking antibody overcomes the resistance mechanism for aPD-1 + MEKi treatment associated with KPC_S tumors at large size.

**Figure 5:**
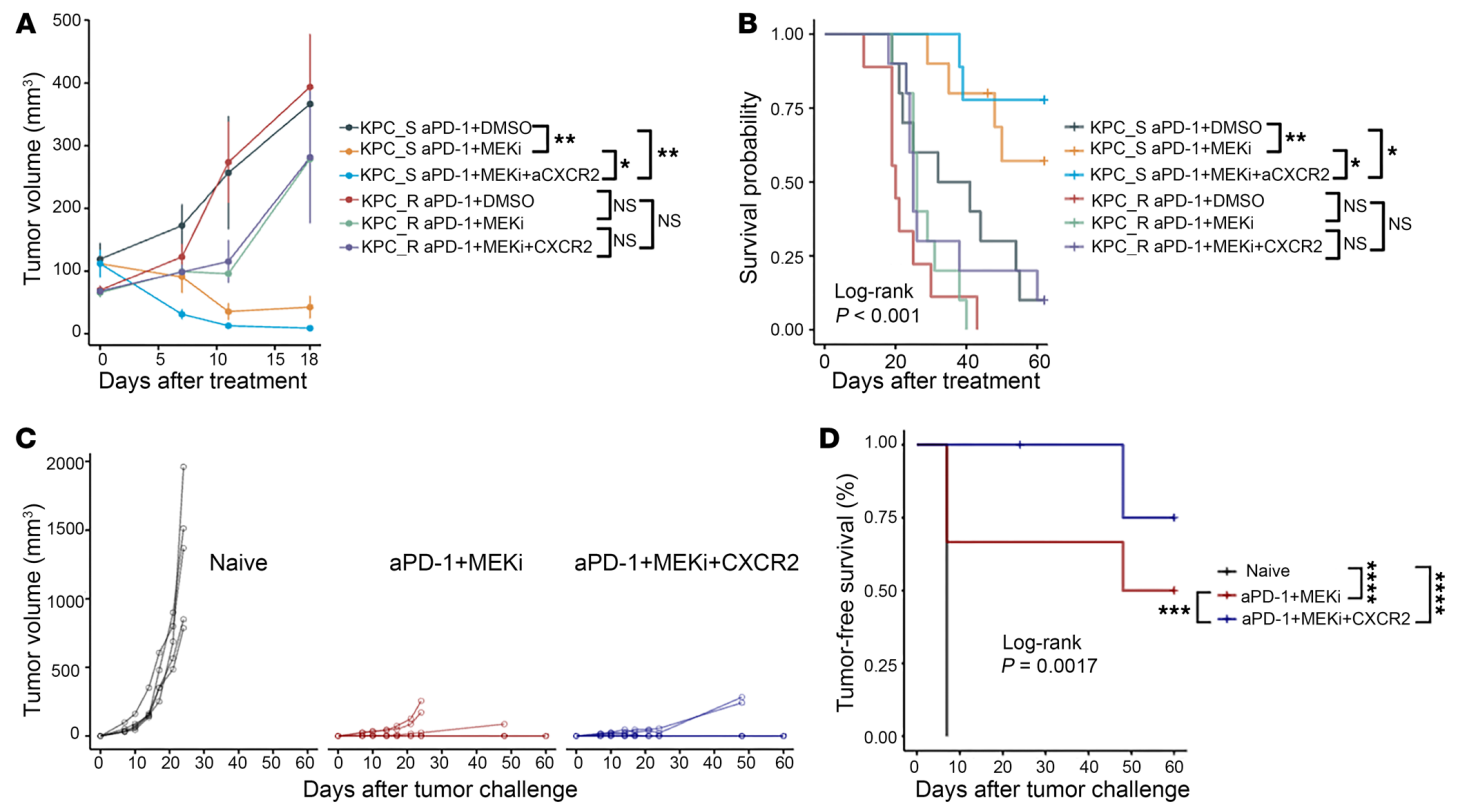
Anti-CXCR2 blocking antibody induces more durable antitumor response following anti-PD-1 + MEKi treatment associated with KPC_S tumors at large size. (**A**) Growth curves of mice treated with MEKi ± aPD-1 ± -aCXCR2, as indicated. KPC_S: KPC001BH; KPC_R: KPC3403F, as indicated here and below. (**B**) Kaplan-Meier survival curves of mice in **A**. (**C**) Caliper-measured tumor volumes of subcutaneously re-challenged tumors. (**D**) Tumor-free survival time in mice following tumor rechallenge. Results are presented as mean ± SEM. Growth curves were compared by the mixed effects model and survival curve compared by the Log-rank test. *P < 0.05; **P < 0.01; ****P < 0.0001.

We next examined whether increased infiltration of TANs and/or CXCR2^+^ TANs accounts for the treatment resistance observed with aPD-1 + MEKi–treated KPC_R tumors. As our previously published PDAC specimen analyses (19) have established the role of targeting CXCR2^+^ TANs in PDAC; this study has, therefore, focused on examining the role of anti-CXCR2 antibody in overcoming the resistance to the combination of aPD-1 and MEKi. Specifically, we assessed the efficacy of adding an anti-mouse CXCR2 blocking antibody (aCXCR2) to aPD-1 + MEKi in treating orthotopically implanted KPC_R tumors (Figure 5 and Supplemental Figure 3). After the implanted tumors were allowed to grow for 7 days, the addition of aCXCR2 did lead to more in-depth tumor regression and significantly improved survival in KPC_S tumors, but it had no effect on KPC_R tumors (Figure 5A). We noted that the sizes of tumors implanted were approximately 2–3 mm in diameter, larger than those of 1–2 mm in diameter in Figure 3A; thus, some tumors at the initial ultrasound measurement were bigger than others. Interestingly, we found that KPC_S tumors that started with a larger baseline tumor volume (Supplemental Figure 3A) were the tumors that developed resistance to aPD-1 + MEKi, whereas only one tumor that started with a smaller baseline tumor volume developed resistance. In contrast, the addition of aCXCR2 improved both local tumor control and overall survival even in KPC_S tumors that started with a larger tumor volume (Supplemental Figure 3A and Figure 5B). These results together support that CXCR2^+^ TAN infiltration is a mechanism of resistance to aPD-1 + MEKi in large KPC_S tumors and in KPC_R tumors.

The increase in the MPEC and central memory CD8^+^ T cells within aPD-1 + MEKi–treated KPC_S tumors (Figure 4G) suggested that these memory T cells could contribute to a durable antitumor response. To test this hypothesis, the survivor mice from the experiment depicted in Figure 5, A and B were rechallenged with subcutaneously implanted tumors formed by KPC_S cells and given the same therapy they received previously following rechallenge (Figure 5C). New C57Bl/6J mice were used as controls. There were no surviving KPC_R mice available for this “rechallenge” experiment. Orthotopic KPC_S tumor–bearing mice that survived following aPD-1 + MEKi or aPD-1 + MEKi + aCXCR2 treatment remained tumor free significantly longer than new mice after subcutaneous injections with KPC_S tumor cells (Figure 5C). In addition, mice that were treated with aPD-1 + MEKi + aCXCR2 remained tumor free longer and survived longer (Figure 5D) than those treated with aPD-1 + MEKi following tumor rechallenge. The reduction in CXCR2^+^ TANs as a result of aCXCR2 treatment may have further augmented the T cell response and subsequent memory T cell formation.

### The effect of aPD-1 + MEKi treatment on NETosis and CD8^+^ T cells in the NETosis areas.

We observed that TANs and CXCR2^+^ TANs infiltrated both KPC-R and KPC_S tumors following aPD-1 + MEKi regardless tumor size. Thus, aPD-1 + MEKi–associated resistance noted in large KPC_S tumors cannot be attributed to either TANs or CXCR2^+^ TANs but to a specific aspect of TANs that are present in large tumors. As large PDAC tumors are known to be associated with hypoxia and necrosis, which were indeed present in the larger KPC_S tumors (Supplemental Figure 4A), we next examined the intracellular signaling associated with hypoxia and necrosis with a focus on the NETosis process. Our hypothesis that NETosis may be associated with aPD-1 + MEKi–treatment resistance in large KPC tumors is further supported by prior reports showing that increased hypoxia can induce NETosis through stabilization of HIF1a signaling within TANs (41).

**Figure 6:**
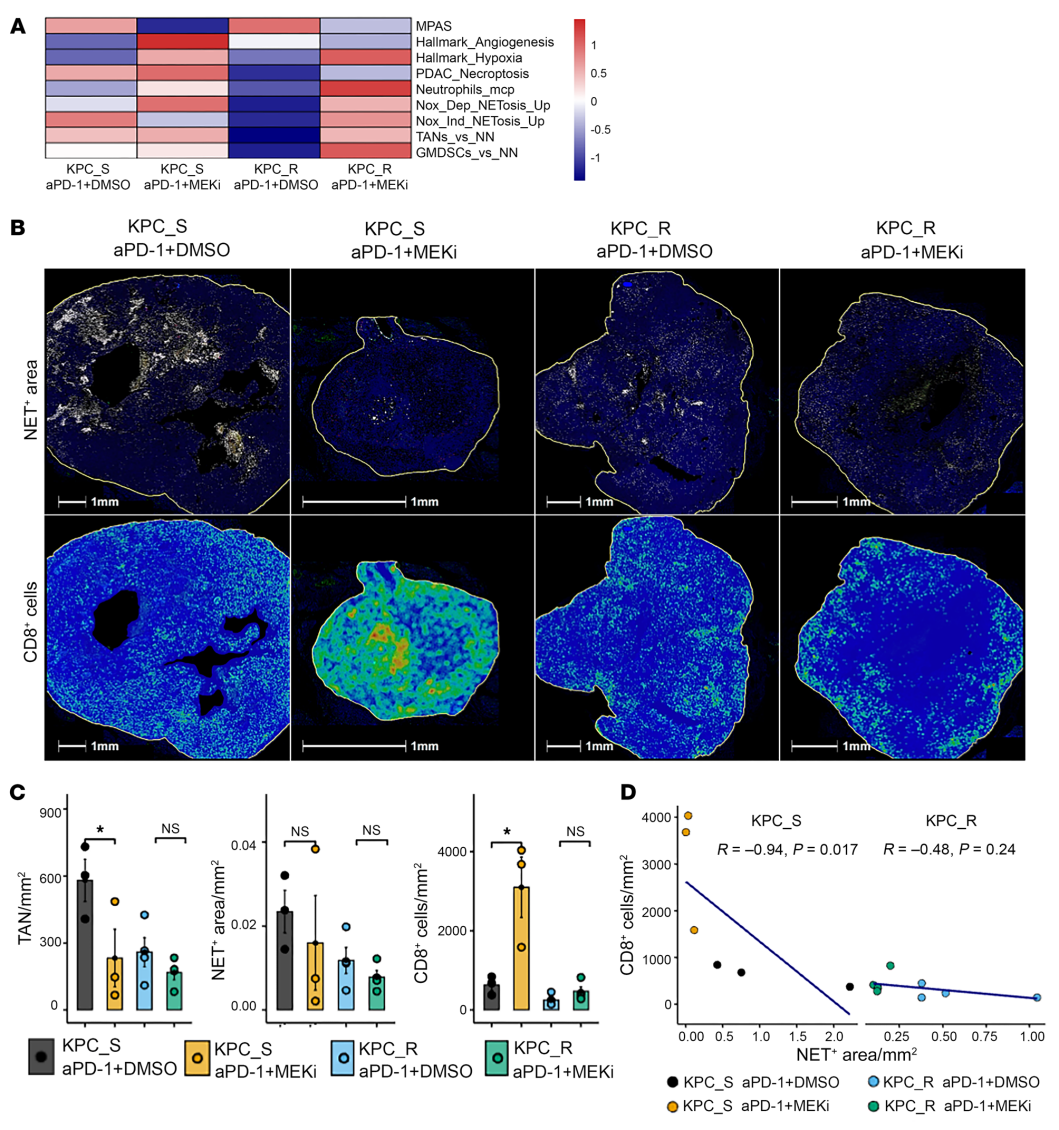
Characterization of NETosis in the PDACs treated by aPD-1 with or without MEKi. (**A**) Singscore for the NETosis gene sets through a pooled bulk-tumor RNA-seq analysis following different treatments, as indicated (*n* = 5 pooled tumors/group). KPC_S: KPC001BH; KPC_R: KPC3403F, as indicated here and below. MPAS, MAPK Pathway Activity Score; Neutrophil_mcp, Neutrophil Microenvironment Cell Populations counter; TAN versus NN, Fold changes of genes upregulated in TANs versus Tissue Naive (circulating) Neutrophil. Necroptosis score was calculated with a panel of genes previously described. (**B**) Multiplexed immunofluorescence staining of NETs and CD8^+^ T cells in the same orthotopic tumors as in **A**. Blue, DAPI; White, MPO; Green, CD8. Note that areas of necrosis are adjacent to the areas enriched with NETosis, but less infiltration of CD8^+^ T cells. Scale bars: 1 mm (note that the sizes of tumors vary due to different treatment effects and, therefore, tumor images were amplified at different scales to similar sizes, resulting in different sizes of 1-mm scale bars). (**C**) Densities of TANs, NETs and CD8^+^ T cells counted in random 20 × HPFs in tumors from different treatment groups, as indicated. Statistics were conducted by Kruskal-Wallis tests without multiple comparisons. *P < 0.05. (**D**) Correlation between CD8^+^ T cell density and NET density. Pearson correlation was conducted with R and *P* value indicated. **B**–**D**, *n* = 3 or 4 mice/group. Experiment was repeated twice.

To test this hypothesis, RNA extracted from bulk tumors of KPC_S and KPC_R orthotopic tumors treated with either aPD-1 + MEKi or aPD-1 + DMSO were pooled and subjected to the whole transcriptomic RNA sequencing (RNA-seq). The resulting Transcripts Per Million–converted (TPM-converted) read counts were run through Singscore for NETosis gene sets (Figure 6A). aPD-1 + MEKi treated tumors clustered together in the hypoxic gene sets, but with marked reduction in the normalized enrichment score (NES) in KPC_S tumors compared with KPC_R tumors. With the NETosis-specific gene sets, Singscore demonstrated a decrease in the NES score of NOX-independent NETosis gene signature in KPC_S tumors, but not KPC_R tumors, following aPD-1 + MEKi treatment. This result suggested that the aPD-1 + MEKi treatment resistance is associated with NETosis.

To directly detect NETs within these tumors and to assess their impact on CD8^+^ T cell infiltration, tumors from mice not used for the immunophenotyping in Figure 4, were stained for markers of NETosis on FFPE tissue sections. NETs can be detected in tissue by colocalizing signals of MPO and DNA, which are separated in non-NET–forming neutrophils (42). Histone H2B could also be used to aid detection while citrullinated histone H3 could be used as a specific marker of NOX-independent NETosis (43). Given the importance of NOX-dependent NETosis to MEK inhibition, we chose not to utilize histone H3. We also found the utility of H2B to be superfluous to DNA and, thus, H2B was dropped from subsequent NET panels. Interestingly, we found that both KPC_S and KPC_R tumors treated with vehicle/isotype controls and KPC_R tumors treated with aPD-1 + MEKi were significantly enriched with NETs (Figure 6, B and C). Areas of necrosis were surrounded by areas enriched with NETs, but scarce of CD8^+^ T cells. Interestingly, excluding necrotic areas, TAN density became smaller following aPD-1 + MEKi treatment compared to aPD-1 + DMSO in KPC_S tumors. This result differs from TAN density per tumor weight (Figure 4B), which appeared to have increased following aPD-1 + DMSO treatment. It is likely because TAN density per tumor weight in the tumors treated with aPD-1 + DMSO were diluted by large necrotic areas (Figure 6B). Note that KPC_R tumors were smaller than large KPC_S tumor; thus, the NET(+) area in KPC_R with aPD-1 + DMSO was not larger than that in KPC_S tumors. In contrast, NET density was not decreased significantly following aPD-1 + MEKi treatment compared with aPD-1 + DMSO in KPC_S tumors (Figure 6C) suggesting that MEKi does not target NETosis and also suggesting an increased ratio of NET to TAN density. NET density in tumors from the aPD-1 + DMSO and aPD-1 + MEKi groups together correlated negatively with CD8^+^ T cell density in the same tumors. This correlation was only statistically significant in the KPC_S tumors, but not in the KPC_R tumors (Figure 6D). Taken together, these results support the hypothesis that NETosis influences the resistance mechanism observed with aPD-1 + MEKi treatment of large, necrotic tumors and that of KPC_R tumors.

### CXCR2 blockade inhibits NETosis by suppressing TAN recruitment to the hypoxic tumor areas.

**Figure 7:**
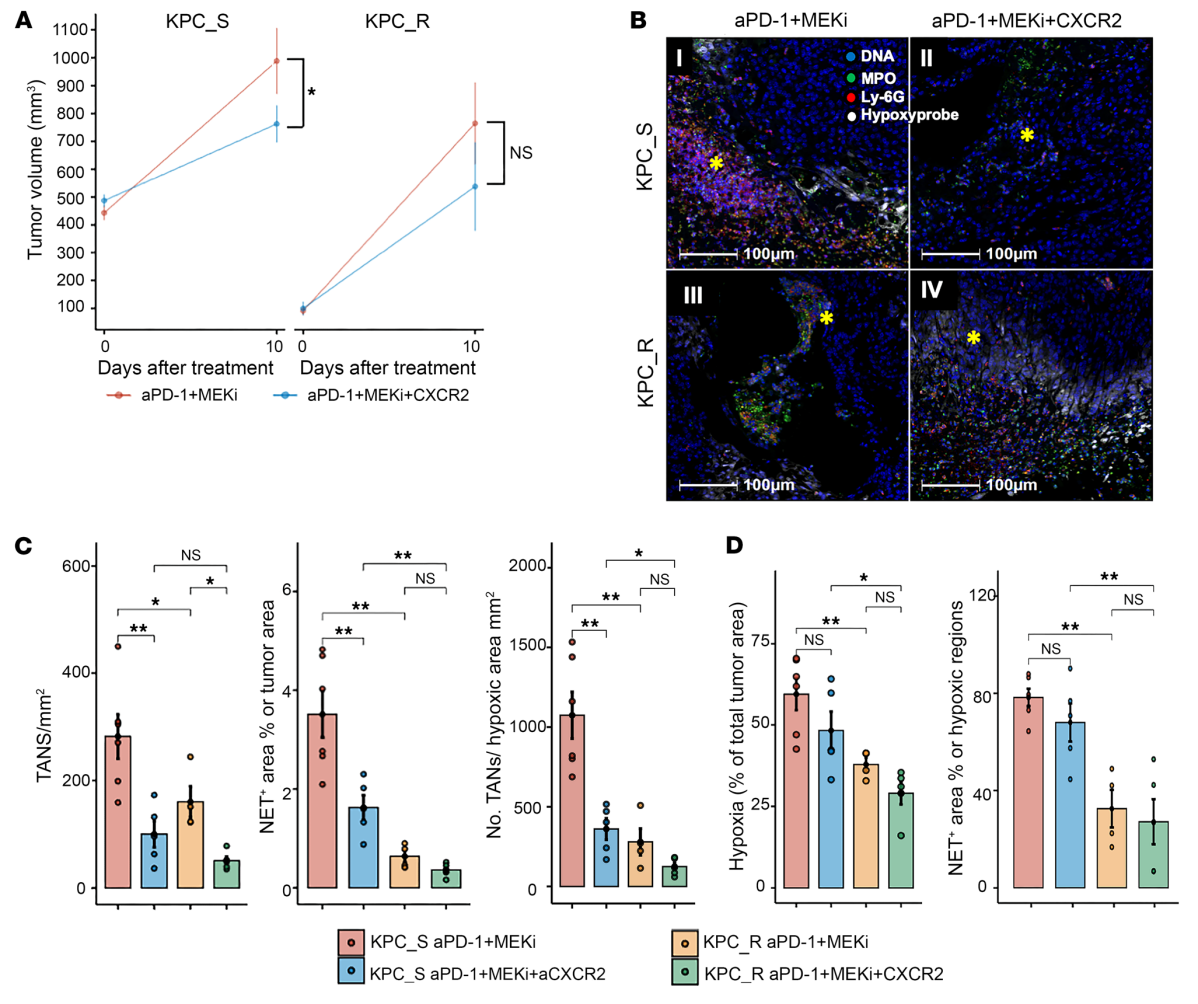
The effect of CXCR2 blockade on NETosis and TAN recruitment to the hypoxic tumor areas. (**A**) Orthotopic tumors were allowed to grow for 21 days after establishment before they were treated with aPD-1 + MEKi or aPD-1 + MEKi + aCXCR2, as indicated, for 10 days (*n* = 4–6 mice/treatment group). Tumor volumes (vol) were measured before treatment as Day 0 and on Day 10. Tumor volumes on Day 10 were compared by the mixed effects model. On Day 10, tumors were harvested for multiplex immunofluorescence analysis in **B**–**D**. (**B**) Representative multiplex immunofluorescence staining images of NETosis at the border of necrotic tumor regions. * indicates NET+ areas. Staining markers are color coded as indicated. Ly-6G+, TAN; Ly-6G+MPO+, NETosis; Hypoxyprobe, hypoxia. An enlarged picture was provided in Supplemental Figure 4. (**C**) Comparison of TAN density and total NET+ area per tumor area and TAN density per hypoxia area between treatment groups. (**D**) Comparison of the percentage of hypoxic area per tumor between treatment groups (left). Comparison of the percentage of total NET+ area within hypoxic regions in same region (right). Results are presented as mean ± SEM. Statistics were performed by Kruskal-Wallis test and multiple comparisons. *P < 0.05; **P < 0.01.

To further assess the role of TANs, NETosis, and hypoxia in mediating the response and resistance to aPD-1 + MEKi treatment, orthotopic tumors at approximately 1–2 mm in diameter were implanted and allowed to grow for 21 days followed by 10 days of treatment. This 21-day growth period was chosen to ensure the tumors were sufficiently advanced and necrotic, which is often found in human metastatic cancers. Mice were injected with Hypoxyprobe 1-hour before tumor harvest to enable quantification and visualization of tumor hypoxia. Hypoxyprobe is a small molecule that forms adducts with protein thiol groups under hypoxic conditions and is used for labeling hypoxic areas with an adduct-specific primary antibody for immunofluorescence staining. We confirmed that KPC_S tumors with a mean pretreatment baseline volume of approximately 500 mm^3^ became sensitive to aPD-1 + MEKi in combination with aCXCR2, whereas KPC_R tumors at the same sizes were resistance to aPD-1 + MEKi in absence of aCXCR2. However, when KPC_R tumors were allowed to grow for 21 days, even though they did not grow up to 500 mm^3^, they still did not share a statistically significant benefit from aCXCR2. Nevertheless, a trend in growth suppression with the addition of aCXCR2 was observed (Figure 7A).

The tumors harvested from this experiment were processed for multiplex immunofluorescence staining of TAN, NETosis, and areas of hypoxia (Figure 7B and Supplemental Figure 4B). When the tumors were larger than 100 mm^3^, there were demonstrable necrotic and hypoxic areas in the tumor compared with their smaller counterparts. The addition of aCXCR2 significantly reduced TAN density in both KPC_S and KPC_R tumors by approximately 4-fold. However, with the addition of aCXCR2, NET density in KPC_S tumors was significantly reduced by approximately 3-fold, whereas NET density was not reduced in KPC_R tumors (Figure 7C). These results suggested that aCXCR2 overcomes resistance to aPD-1 + MEKi mediated by TANs or TAN-associated NETosis in large KPC_S tumors and that KPC_R tumors appear to have an additional resistance mechanism for aCXCR2.

Hypoxia area as percent of total tumor area was significantly different between KPC_S and KPC_R tumors but was not significantly changed following the addition of aCXCR2 treatment (Figure 7D). In contrast, the density of TANs within the hypoxic areas was significantly reduced following aCXCR2 treatment (Figure 7C). To assess if aCXCR2 blockade directly altered NETosis, we quantitated the NET(+) area within the hypoxic area. We did not notice any significant change in NET(+) area following the addition of aCXCR2 treatment, suggesting that NETosis was reduced by suppressing TAN recruitment via CXCR2 blockade (Figure 7D). In addition, these data suggest that NETosis is produced from TANs that are recruited to the necrotic/hypoxic tumor areas and CXCR2 blockade can suppress NETosis by blocking the recruitment of TANs to hypoxic areas.

### A risk score driven by the interaction between NETosis and MAPK signaling is significantly associated with poorer survival in human PDACs.

**Figure 8:**
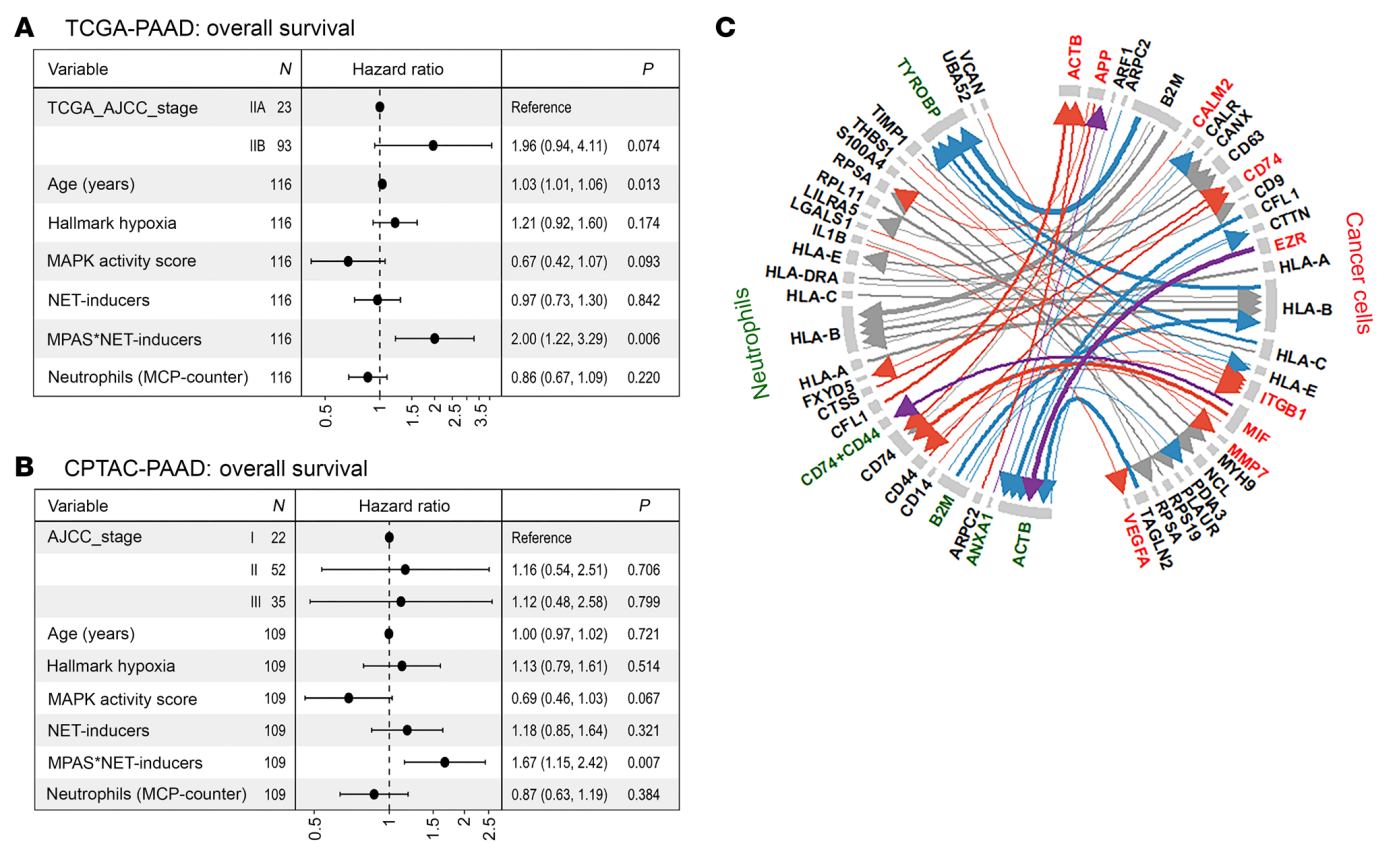
Multivariate cox-hazard model for assessing the survival correlation of MAPK and NETosis gene signatures in TCGA and CPTAC PDAC datasets and single cell analysis for assessing the interaction between NETosis and MAPK pathways. (**A**) TCGA; (**B**) CPTAC. MPAS, MAPK Activity Score. NET inducer, NETosis-inducing gene set. MCP Counter, Microenvironment Cell Populations counter. Reference: hazard ratio and 95% confidential interval. (**C**) The interaction between PDAC cancer cells and neutrophils. The clusters of PDAC cells and neutrophils were identified by single cell analysis. The top 50 strong interactions mediated by the genes involved in MAPK pathways (in red) in PDAC cells and those in the NET/NETosis pathways (in green) in neutrophils, respectively, as indicated, were delineated by arrows. Blue arrows delineate the interactions involving the genes in the NET/NETosis pathways; red arrows delineate the interactions involving the genes in the MAPK pathways; and purple arrows delineate the interactions involving genes in both MAPK and NET/NETosis pathways.

We next assessed the relevance of NETosis with human PDACs by analyzing the data in the TCGA (Supplemental Table 2) and CPTAC database (Supplemental Table 3). We chose both databases to validate the results by each other, although it should be noted that the data quality and distribution of cases among different stages of PDAC vary between the 2 databases (Figure 8A) and that both databases mainly include stage I–III PDACs. We chose to use both datasets as independent validators of each other. The TCGA dataset was limited to stage I and II after applying a quality control filter of survival data. Due to the MEK inhibitors targeting of the mutant KRAS pathway, the MAPK activation signaling gene signature (44) was included in the analysis in addition to the NETosis-inducing gene sets (45). Using the tumor purity–normalized TCGA and CPTAC human PDAC RNA sequencing data sets, we employed a single-sample gene set enrichment assay to analyze the hypoxia signature, which was derived from the MSigDB Hallmark Hypoxia pathway, the MAPK activity score (MPAS) (44), the NETosis-inducing gene set (NET inducer), and the Neutrophil Abundance, by using the Microenvironment Cell Populations counter (MCP-Counter) (46). After filtering PDAC cases for survival beyond 90 days to exclude those who may have died from perioperative complications, a Cox-proportional hazard model was run for each dataset, respectively.

We did not find any of the above factors of hypoxia, MPAS, NET inducer, or neutrophil abundance to be prognostic in the univariant analysis (Supplemental Tables 4 and 5). Higher tumor stage, older age, and increased hypoxia shared trends associated with inferior survival. Interestingly, MPAS is associated with a strong trend toward improved survival in the multivariant analysis, suggesting that it may interact with other factors. We also noted that MPAS and NETosis-inducing gene sets were not 2 independent prognostic factors (Supplemental Figure 5) but instead, the prognostic value of MPAS was influenced by the expression of the NETosis gene sets, particularly in the TCGA dataset. A combined interaction plot and risk probability showed that PDACs with low MPAS and high NET-inducer score were at the lowest risk (best prognosis), while high MPAS and high NET-inducer scores were associated with the highest risk (worst prognosis). This is consistent with our above results (Figure 4B) showing that aPD-1 + MEKi treatment of smaller-size, MEKi-sensitive murine tumors, was associated with increased TAN infiltration, which would be anticipated to be associated with higher expression of NET-inducing genes. Together, these results suggested that MAPK signaling or NETosis-inducing gene by itself is not a poor prognostic factor. Therefore, we developed a risk score that is driven by the interaction between the NETosis-inducing gene sets and MPAS (designated MPAS*NET-inducers). After correcting for age and clinical stage, the risk score driven by the interaction between MPAS and NET inducer scores was strongly correlated with worse prognosis (HR: 1.99, 1.22–3.55; *P* = 0.006) for both TCGA and (HR: 1.67, 1.15-2.42, *P* = 0.007) CPTAC cohorts respectively (Figure 8, A and B). Spearman’s correlation showed that hypoxia scores correlate positively with NET inducer scores and also with the *CXCL8 (IL8)* expression (Supplemental Figure 6). The single cell suspension from a total of 15 resected PDAC tumors were clustered into multiple cell types. Ductal epithelial cells, which were anticipated to be primarily PDAC cells, as well as the cell types in the TME, were identified according to the markers previously established (47). The myeloid cell population was further clustered into myeloid subtypes, including macrophages, dendritic cells, and neutrophils. The cell-to-cell interaction between PDAC cells and neutrophils was analyzed (Supplemental Figure 6). Genes that mediate this cell-to-cell interaction are enriched with those in both the MAPK and NET/NETosis pathways according to the GSEA analysis. GO were used to further identify the genes whose products are functionally involved in the MAPK pathways in PDAC cells and in the NET/NETosis pathways in neutrophils, respectively (Supplemental Data Set 1). The results showed that the top 50 strong interactions were mediated mostly by the genes involved in MAPK pathways in PDAC cells and those in the NET/NETosis pathways in neutrophils (Figure 8C). Together with the literature showing that hypoxia induces MAPK signaling, these results suggested that hypoxia also upregulates NETosis-inducing genes, including *CXCL8,* which subsequently skews the neutrophil response towards NETosis.

## Discussion

To our knowledge, this study is the first to describe that hypoxia/necrosis and its induced NETosis serve as mechanisms that mediate resistance to aPD-1 and MEK inhibition treatment in PDAC. We found that the combined aPD-1 and MEK inhibition can completely eliminate orthotopically implanted, small-size tumors formed by mouse PDAC cell lines. This combination is also effective in eliminating metastatic PDAC in a hemispleen mouse model of metastasis. Moreover, this combination treatment confers increased effector CD8^+^ T cell response, as evidenced by increased cytotoxic and memory T cell subtypes. However, this study showed that large-size tumors remain resistant to combined aPD-1 and MEK inhibition due to NETosis that is formed from treatment-induced TANs. Nevertheless, CXCR2 blockade can suppress NETosis by blocking recruitment of TANs to hypoxic areas and resensitize tumors to combined aPD-1 + MEKi, providing a mechanistic rationale for the clinical development of CXCR2-targeting combination therapies.

In this study, a pancreatic cancer treatment resistance mechanism was linked to the size of the tumors and hypoxia/necrosis that is associated with large-size tumors. However, the observed signaling of the MAPK pathway alone and measurement of NETosis were not by themselves predictive of resistance to MEKi. Instead, a risk score driven by the interaction between the MAPK signaling and NET-inducing genes strongly correlated with worse prognosis in patients with PDAC. Our results suggest that it was the quantity of TANs that subsequently determined quantity of NETs in the TME. Therefore, any component of NETs may play a role in impeding T cell trafficking or function. The precise molecules, such as CitH3 (48), will be used to determine NETs formation in the future studies.

Our study also showed that combined aPD-1 and MEKi induce TAN infiltration into PDACs and may be an indicator of response in small tumors responding to the treatment. Our results support the notion that more established, large tumors contain higher TAN density with a possibly shift toward a protumoral N2 polarization. As such, the observed MEKi-induced recruitment of neutrophils in smaller tumors likely represents an initial increase in antitumor N1-like TANs, which warrant a direct analysis in the future studies. This study provides the rational for testing CXCR2 blockade together with MEKi, particularly to block NETosis in larger disease burden situations to overcome the resistance to aPD-1 therapies. It is thus important to establish a direct NETosis relationship in future preclinical studies by using DNase I or PAD4 inhibitors (49–53). As our previously published PDAC specimen analyses have established the role of targeting CXCR2^+^ TANs in PDAC, this study has, therefore, focused on examining the role of anti-CXCR2 antibody in targeting NETosis to overcome the resistance to the combination of aPD-1 and MEKi. In this study, we did not test the combination of MEKi and anti-CXCR2 antibody alone because this study was initiated to identify the Kras-downstream effector that may be targeted to sensitize PDACs for aPD-1 therapy. Nevertheless, it remains possible that CXCR2 blockade can overcome resistance to MEKi as single agents by using the same mechanism demonstrated in this study.

This study was initiated to identify therapies that can overcome resistance to Kras signaling in PDACs because clinical trials testing the combination of aPD-1 and MEKi showed few responses in patients with PDAC and in patients with other gastrointestinal malignancies (54). The patients enrolled in these clinical trials had metastatic PDACs and generally had large disease burdens. Their prior treatments may have also induced hypoxia signals and subsequent NETosis at the time when their cancers became refractory to prior treatments and before enrollment into MEKi clinical trials. This study may provide a mechanistic clue for the poor response of these patients to MEKi. One limitation of this study is the lack of validation of this resistance mechanism through clinical trials due to the lack of availability tumor specimens. However, human PDAC database analysis provided evidence of human correlation to support further clinical testing of inhibitors of NETosis. This mechanism may be particularly applicable for understanding early resistance to the new class of mutated KRAS inhibitors that emerged after we have initiated this study and shall be examined in the preclinical studies. Studies designed to test combinations of MEKi or KRAS inhibitors and NETosis inhibitors and obtain serial biopsies in responders and nonresponders are needed to further analyze the role of NETosis as a KRAS pathway–mediated resistance mechanism.

The findings in this study are limited to orthotopic and hemisplenic models of KPC tumors. The genetically engineered KPC mouse model was not used due to the difficulty in controlling the tumor size and burden. In this study, KPC_R tumors were primarily used for validating the NETosis-mediated resistance mechanism; however, we did not address the mechanism underlying the lack of the same level of aCXCR2 sensitivity in KPC_R tumors compared with the large KPC_S tumors. There are clear baseline differences in T cell infiltration between the KPC_S and KPC_R tumor models that could by themselves explain the apparent different sensitivity to the aPD1+MEKi treatment. KPC_R tumors showed low effector memory T cells and IFN-γ^+^ CD8^+^ T cells in the mice treated by aPD-1 + MEKi (Figure 4), suggesting that the resistant mechanism leads to the failure of the induction of cytotoxic effector T cells in KPC_R tumors following aPD-1 + MEKi. Such a mechanism could be a primary one underlying the resistance in KPC_R tumors to aPD-1 + MEKi. KPC_R tumors have smaller percentages of hypoxia areas compared with KPC_S tumors and subsequently smaller percentages of NETosis areas (Figure 7D), suggesting that the hypoxia-NETosis axis plays a minor role in the treatment resistance in the KPC_R tumors. On another hand, TANs remain low following the aPD-1 or aPD-1 + MEKi treatment (Figure 6), suggesting that the resistance mechanism is associated with other components in the TME such as macrophages or fibroblasts. The rapid reduction in tumor burden in sensitive tumors impeded RNA-seq analyses of isolated neutrophils and thus our molecular profiling was limited to the bulk-tumor level. Repeat experiments using a more appropriate technology such as single-cell RNA-seq will complement our findings by providing a neutrophil-specific dataset for between-treatment comparison when the neutrophil population can be more reliably identified in PDAC tumors in the future.

Currently, the combination of anti-PD-1 antibody and various CXCR2-targeting agents have been tested in solid tumors. Previously, such a combination has been examined in patients with metastatic melanoma whose diseases have progressed through either anti-PD-1 antibody or the combination of anti-PD-1 antibody and anti-CTLA-4 antibody in the past, resulting in an overall objective response rate of 21% (55). The combination has been well tolerated; and thus, a similar strategy combining anti-PD-1 antibody tislelizumab and CXCR1/2 inhibitor SX-682 is being tested by our group in the neoadjuvant clinical trial for resectable PDAC (NCT05604560). Analysis of the specimens obtained from this neoadjuvant clinical trial will further provide the rationale for testing the triple combination of aPD-1, MEK inhibitors, and CXCR2 inhibitors in future clinical trials.

## Methods

### Sex as a biological variable.

Female C57BL/6J mice were used as hosts of syngeneic tumor cell lines that all happen to have been derived from female mice, although the results are likely to be applicable to male mice as well.

### Cell lines and cell culture.

The *Kras*^G12D^ *p53 PDX1-Cre* (KPC) cell lines used in this study were derived from the KPC mouse model of PDAC as previously described (32, 33). Cells were grown in RPMI1640 medium (Life Technologies) supplemented with fetal bovine serum (FBS, Benchmark), 2 mM L-glutamine (Life Technologies), 1% MEM Non-Essential Amino Acids Solution (MEM-NEAA, Life Technologies), 1% sodium pyruvate (Sigma), 1% Penicillin/Streptomycin (pen/strep, Life Technologies), 0.2 Units/ml Regular Human Insulin (2 ml) (Novo Nordisk) at 37°C with 5% CO_2_.

### Inhibitors and reagents.

Trametinib (GSK1120212) was obtained from SelleckChem (Cat# S2673) and resuspended in DMSO and frozen at –20°C at the start of each dosing experiment. Mice were dosed daily for the length described in each experimental section. Dosing was performed via intraperitoneal injection at a final concentration of 0.5 mg/kg assuming average mouse weight of 20 grams. aPD-1, aCXCR2 and IgG/isotype control antibodies were provided by Bristol-Meyers Squibb. aPD-1 and its isotype control were given intraperitoneally (i.p.) at 100 mg twice a week; aCXCR2 and its isotype control were given i.p. at 250 mg twice a week.

### Mouse studies.

Female C57BL/6J mice were purchased from Jackson Laboratories (Bar Harbor, ME), habituated to mouse colony for 1–2 weeks, and used at 10–12 weeks of age. Orthotopic tumor models were modified from our previous reports (32). Briefly, 1–2 × 10^6^ PDAC cells of the KPC cell lines were subcutaneously injected into the flanks of syngeneic female C57BL/6J mice under isoflurane. At 10–14 days post-injection, the subcutaneous tumors were harvested and cut into approximately 2 mm^3^ pieces, and surgically implanted into recipient C57BL/6J mice. Tumors were monitored via ultrasound using a Vevo770 (Visualsonics Inc., Toronto, Canada) and tumor volume was calculated using the formula for ellipsoid objects 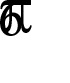
× (*L* × *W* × *H*). Tumors were allowed to grow for 3, 7, 14, or 21 days prior to treatment for each specified experimental condition. Importantly, mice were randomized into treatment groups using baseline tumor volume and body weight values prior to treatment. Randomization was achieved using the R package HAMLET with dummy mice for uneven grouping conditions.

Hemi-spleen tumors were done as previously described (35). Briefly, KPC cells were harvested at 40%–70% confluency, spun down and washed, and resuspended to 2.5 × 10^6^ cells/ml for KPC and 20 × 10^6^ cells/ml for Panc02 cells in hemi-spleen buffer (HBSS, Life Technologies) with 1:2000 Anti-Clumping Agent (Gibco) to prevent aggregation of cells prior to injection. Injections were prepared by withdrawing 150 μl of sterile PBS followed by 100 μl of cell suspension. Liver metastases were allowed to grow for 7–10 days prior to treatment.

### RT-qPCR.

Mice were treated for 7 days with controls or treatments and tumor was harvested and processed for RNA extraction using the Qiagen DNA/RNA/Protein Kit. cDNA was produced from 1 μg of RNA using the ReadyScript cDNA Synthesis Mix (Sigma #RDRT) and 50 ng of cDNA was used for RT-qPCR with the PowerUp SYBR Green Master Mix (Thermo-Fisher Sci.) on an Applied Biosystems StepOnePlus machine. Relative quantification was performed in the StepOne Software v 2.3 using beta-actin as control gene and IgG + DMSO as reference group. Purity and quantity of RNA and cDNA were determined by fluorometrically using the Qubit BR RNA Assay and ssDNA assay respectively. Samples were pooled prior to performing qPCR. Primers were obtained from RealTimePrimers Mouse Cytokine I & II primer sets.

### In-cell Western blot.

KPC cells were seeded at 1 × 10^4^ cells/well in duplicate in a 96-well plate and allowed to grow for one day prior to administration of serially-diluted MEKi for 24 hours. Cells were washed with PBS and fixed in 10% NBF for 20 min at 4°C followed by permeabilization in 5 washes with 0.1 % Triton X-100 in PBS. Wells were blocked with LI-COR blocking buffer and probed for pMek1/2 (Cell-Signaling Technologies #9121) and pErk1/2 (Cell-Signaling Technologies #4370) for 2 hours at RT. Detection was performed with IRDye 800CW (LI-COR) conjugated secondary antibodies for 2 hours at room temperature and total signal was normalized to cellular abundance using CellTag700 (LI-COR) to account for differential cellularity between wells. Imaging and analysis was performed on LI-COR Odyssey Scanner and Empiria Studio 2.2 software respectively.

### TILs isolation & flow cytometry.

Orthotopic pancreatic tumors were isolated, minced, and digested with Tumor Dissociation Kit (Miltenyi Biotec) 3 days after the last treatment. Tissues of each tumor was dissociated using the gentleMACS Octo Dissociator (Miltenyi Biotec) with a heater-run program at 37°C. Dissociated tissues were washed with fresh media, and passed through a 100-μm and 40-μm nylon filters sequentially to acquire a single-cell suspension and brought to a volume of 20 mL in CTL medium (RPMI 1640 media (Life Technologies) supplemented with 10% heat-inactivated fetal bovine serum (HI-FBS, Benchmark), 1% penicillin/streptomycin (pen/strep, Life Technologies), 1% HEPES (Life Technologies), 1% MEM Non-Essential Amino Acids Solution (MEM-NEAA, Life Technologies), 1% L-glutamine (Life Technologies), and 0.05 mM 2-mercaptoethanol (Sigma)). The red blood cell lysis was conducted by using ACK lysis (Quality Biological). Cell pellets were then washed twice and resuspended in 6 mL of 80% Percoll (GE Healthcare LifeSciences), overlaid with 6 mL of 40% Percoll, and centrifuged at room temperature for 25 minutes at 3,200 rpm without brake. The leukocyte layer was removed and quenched with 30 mL of CTL media.

Following the isolation of TILs from the murine pancreatic tumor, samples were washed with PBS and processed for live/dead cell discrimination using LIVE/DEAD Fixable dyes (Invitrogen). All panels are included in Supplemental Tables 6–8. TILs were washed and subsequently blocked with Fc antibody (BD Pharmingen) for 10 minutes on ice, followed by staining with following cell surface antibodies (Supplemental Tables 6–8) for 60 minutes on ice. Flow cytometry was performed using CytoFLEX (Beckman Coulter). Flow data were analyzed using CytExpert software (Beckman Coulter). All data was analyzed in R.

### Multiplex immunofluorescence.

3 μm sections of FFPE tissue were processed by routine IHC protocol. Briefly, slides were de-waxed and re-hydrated and heat-induced antigen retrieval was performed for 20 minutes in a vegetable steamer. Slides were allowed to cool to room temperature in a fume hood, washed 2 times in 5X TBS-T and stained for 1 hour with primary antibodies to Ly-6G, CD8, CD66b, H2-B and Hoechst 3342 all at 1:100 dilution. Following 2 x 5’ washing, secondary antibodies were applied for 2 hours at RT and washed 2 x 5’. Slides were mounted with Vectashield and scanned on a Polaris scanner. Entire tumor area was processed for background/AF subtraction in “inForm” and analyzed in HALO using the AreaQuant and HiPlefFL modules. Results were exported and analyzed in R.

### RNA-seq analysis.

Mice were treated for 7 days before tumor was harvested and processed for RNA extraction using the Qiagen DNA/RNA/Protein Kit. 250 ng of RNA from 5 mice per group was pooled and sequenced by BGI Genomics, Inc. which also performed QC/QA analyses. Raw counts were aligned to mm10 genome in R using the ‘Rsubread’ package. Raw counts were first normalized by gene length to obtain reads-per-kilobase (RPK) and then normalized by TMM and filtered by expression via the edgeR package following the outline for GeTMM methods (56) in The R package Sinscore was used to measure single-sample gene set enrichment scores against the murine MSigDB (57) collections and custom gene sets as described in the results section. Imputation of immune cell types was performed using MCP-Counter package for mouse (58).

### TCGA/CPTAC analyses.

TCGA data was obtained from TCGABiolinks R package (59). STAR counts were first normalized by gene length to obtain reads-per-kilobase (RPK) and then normalized by TMM and filtered by expression via the edgeR package following the outline for GeTMM methods (56). Patients were subset to the original 150 patients analyzed in the TCGA PAAD paper to ensure high-quality patient samples were used. The Singscore package was used to derive per-sample gene set scores. Importantly, updated survival information was obtained from cBioportal TCGA-PAAD annotations. Survival analysis was performed using the Survminer package after filtering out any patient with a less than 90 day survival. Immune cell abundance was derived with the MCP-Counter R package. All gene set, RNA-seq, and associated continuous data were transformed to Z-scores prior to analysis, as many variables were of different scales. Imputation of immune cell types was performed using MCP-Counter package (58) for human data.

Variance inflation diagnostics were confirmed to be low/absent prior to Cox modeling. Cox proportional hazards formula was: coxph(Surv(survival_time, survival_status) ~ TCGA_AJCC_Stage + Age + HALLMARK_HYPOXIA + MPAS * NET_Inducers + Neutrophils_MCP). Analysis of CPTAC data was generally the same excepting that RNA-seq and gene-level protein data was obtained prenormalized from CDC Data Commons.

### Single-cell RNA-seq data processing.

As we described previously (47), raw gene expression matrices were imported into the R statistical environment (v4.5.0) and a Seurat object was initialized using Seurat (v5.0). Quality control and data normalization were also described previously. The data were then scaled using ScaleData, and principal component analysis (PCA) was performed. The optimal number of principal components (PCs) for downstream analysis was determined by inspecting the Elbow plot. For this study, the published dataset was directly used with modified downstream analyses. A total of 117,123 cells derived from 15 clinical samples were retained for analysis. For visualization and clustering, the first 15 PCs were used. Non-linear dimensional reduction was performed using Uniform Manifold Approximation and Projection (UMAP).

### Intercellular communication analysis.

To systematically characterize cell-cell interactions between Cancer cells and Neutrophils, we performed ligand-receptor interaction analysis using CellChat (v1.6.1) (60). To ensure comprehensive coverage, we integrated ligand-receptor pairs from four databases: NATMI, SingleCellSignalR, OmniPath, and CellChatDB. Statistically significant interactions were identified based on a *P*-value < 0.05. The top 50 statistically significant interactions, ranked by communication probability, were selected for visualization. Circle plots and other visualizations were generated using the iTALK (v0.1.0) and circlize (v0.4.16) R packages to illustrate the landscape of intercellular communication. NETosis and MAPK-related genes were identified by KEGG and GO enrichment analysis in all the genes involved in intercellular communications with a *P*-value < 0.05.

### Statistics.

Assessment of in vivo antitumor efficacy was determined by mixed-effects model using the general formula: [Endpoint_Tumor_Volume ~ Treatment*Time + (0 + 1/Mouse)] per KPC line with the lme4, lmerTest, emmeans, and Rstatix R packages with or without appropriate multiple-comparison corrections, as indicated. Comparisons between groups in single-factor dimensions was performed using nonparametric Kruskal-Wallis test followed by multiple comparisons with Tukey’s correction. Correlations were performed using Spearman’s method.

### Study approval.

All animal experiments conformed to the guidelines of the Animal Care and Use Committee of the Johns Hopkins University, and animals were maintained in accordance with the Institutional Animal Care and Use Committee (IACUC) guidelines.

### Data availability.

The single cell RNA-seq data accession number in the GEO database is GSE279781. Any additional information required to reanalyze the data reported in this work paper is available from the lead contact upon request. Values for all data points in graphs are reported in the Supporting Data Values file.

## Author contributions

Concept was conceived by LZ. The strategy and the overall study were designed by BH and LZ. Experiments were conducted by BH and AB. Data were collected by BH. Formal analysis was conducted by BH, YL, and LZ. Original draft manuscript was written by BH. Manuscript was reviewed and revised was by LZ and EMJ. Supervision was made by LZ. The project administrator is LZ.

## Conflict of interest

LZ received grant support from Bristol-Meyer Squibb, Merck, Abmeta, Astrazeneca, Biosion, and Ipsen. LZ was a paid consultant/Advisory Board Member at Biosion, Alphamab, NovaRock, Ambrx, Akrevia/Xilio, QED, Amberstone Biosciences, Tavotek Lab, ClinicalTrial Options, LLC, Histosonics, Pfizer, Fortress, Tallac and Fortvita. LZ holds shares at Alphamab, Cellaration, Amberstone, and Mingruizhiyao. EMJ reports other support from Abmeta and Adventris, personal fees from Achilles, Dragonfly, CPRIT, HDTbio, Mestag, The Medical Home Group, and Surgtx, other support from Parker Institute, grants and other support from the Lustgarten Foundation, Genentech, BMS, and Break Through Cancer outside the submitted work. EMJ is the Dana and Albert “Cubby” Broccoli Professor of Oncology.

## Funding support

This work is the result of NIH funding, in whole or in part, and is subject to the NIH Public Access Policy. Through acceptance of this federal funding, the NIH has been given a right to make the work publicly available in PubMed Central.

- NIH Grant R01 CA169702 (to LZ).
- NIH Grant R01 CA197296 (to LZ and EMJ).
- NIH SPORE Grant P50 CA062924 (to LZ and EMJ).
- NIH Cancer Center Support Grant P30 CA006973 (to LZ).
- NIH Cancer Center Support Grant P30CA054174 (to LZ).
- NCI T32 grant CA126607 (to AB).

## Supplementary Material

Supplemental data

Supplemental data set 1

Supporting data values

## Version 1 03/19/2026

In-Press Preview

## Version 2 05/15/2026

Electronic publication
