# Supplementary material for: CXCR2 blockade overcomes the NETosis-mediated resistance to MEK inhibition in pancreatic cancer models
Source: J Clin Invest. 2026 Mar 19;136(10):e196622. doi: 10.1172/JCI196622 (PMC13178650; doi:10.1172/JCI196622)
Supplement: Supplemental data [file jci-136-196622-s254.pdf]

Supplemental Table S1: Inhibitors of KRAS Signaling

Compound	Target	Name	Dose	Units	Route	Freq	Vehicle	Vendor	Catalog #
KRX-0401	Akt	Perifosine	25	mg/kg	IP	q.d.	PBS	Sigma-Aldrich	SML0612-10MG
BMS	IgG		5	mg/kg	IP	q.d.	PBS	*provided by BMS*	NA
INCB018424	JAK1/2	Ruxolitinib	75	mg/kg	IP	b.d.	DMSO;PBS	ApexBio	A3012
GSK1120212	MEK1/2	Trametinib	0.5	mg/kg	IP	q.d.	DMSO;PBS	Selleck Chemicals	S2673
BMS	PD-1		5	mg/kg	IP	q.d.	PBS	*provided by BMS*	NA
GDC-0941	PI3Kα/δ	Pictilisib	50	mg/kg	IP	q.d.	DMSO;PBS	ApexBio	A8210
RBC8	RALA/B		50	mg/kg	Oral	q.d.	DMSO;PBS	Selleck Chemicals	S7606
NVP-LDE225	SHH	Erismodegib	50	mg/kg	Oral	q.d.	DMSO;PBS	*provided by Novartis*	NA
JHH-048	Yap1			mg/kg	IP	q.d.	Corn Oil	*Liu Lab (Hopkins)*	NA

Supplemental Table S2: Clinicopathologic Characteristics of the TCGA Cohort.

TCGA Cohort: Baseline Characteristics

Characteristic	Composite Risk Low, N = 58	Composite Risk High, N = 58
Age (yrs.), Median (IQR)	61 (53 – 68)	72 (63 – 77)
Primary Dx., n (%)		
Adenosquamous	2 (3.4)	1 (1.7)
Colloid (mucinous noncystic)	0 (0)	1 (1.7)
Ductal adenocarcinoma	53 (91)	55 (95)
Other	3 (5.2)	1 (1.7)
AJCC Stage, n (%)		
IIA	20 (34)	3 (5.2)
IIB	38 (66)	55 (95)
Tumor Purity, n (%)		
High	27 (47)	32 (55)
Low	31 (53)	26 (45)
KRAS Status, n (%)		
Wt	4 (6.9)	4 (6.9)
Mut	54 (93)	54 (93)
OS (months), Median (IQR)	16 (12 – 23)	13 (8 – 20)
OS Status, n (%)		
Alive	33 (57)	16 (28)
Deceased	25 (43)	42 (72)

Supplemental Table S3: Clinicopathologic Characteristics of the CPTAC Cohort.

CPTAC Cohort Baseline Characteristics

Characteristic	Composite Risk Low, N = 55	Composite Risk High, N = 54
Age (yrs.), Median (IQR)	67 (63 – 72)	62 (51 – 69)
Primary Dx., n (%)		
Adenosquamous carcinoma	1 (1.8)	3 (5.6)
PDAC	54 (98)	51 (94)
AJCC Stage, n (%)		
IA	4 (7.3)	3 (5.6)
IB	13 (24)	2 (3.7)
IIA	2 (3.6)	4 (7.4)
IIB	19 (35)	27 (50)
III	17 (31)	18 (33)
Tumor Purity, n (%)		
Low	38 (69)	21 (39)
High	17 (31)	33 (61)
KRAS Status, n (%)		
Wt	1 (1.8)	1 (1.9)
Mut	54 (98)	53 (98)
OS (months), Median (IQR)	20 (12 – 23)	14 (10 – 24)
OS Status, n (%)		
Alive	34 (62)	18 (33)
Deceased	21 (38)	36 (67)

Supplemental Table S4: Combined results of univariate and multivariate Cox proportional hazards models for the TCGA cohort.

TCGA: Cox Proportional Hazard

	Univariate					Multivariate			
Characteristic	N	HR	95% CI	p-value	q-value	HR	95% CI	p-value	q-value
**AJCC Stage**	116			0.14	0.24			0.059	0.14
IIA		—	—			—	—		
IIB		1.62	0.82, 3.19			1.96	0.94, 4.11		
**Age (years)**	116	1.02	1.00, 1.04	0.090	0.21	1.03	1.01, 1.06	**0.011**	**0.040**
**Hallmark Hypoxia**	116	1.23	0.97, 1.55	0.084	0.21	1.21	0.92, 1.60	0.2	0.2
**MAPK Activity Score**	116	1.16	0.90, 1.50	0.25	0.35	0.67	0.42, 1.07	0.089	0.2
**NET-Inducers**	116	1.06	0.86, 1.32	0.58	0.58	0.97	0.73, 1.30	0.8	0.8
**MPAS*NET-Inducers**	116	1.35	1.04, 1.76	**0.027**	0.19	2.00	1.22, 3.29	**0.005**	**0.032**
**Neutrophils (MCP-Counter)**	116	0.93	0.75, 1.14	0.48	0.56	0.86	0.67, 1.09	0.2	0.3

[1] HR = Hazard Ratio, CI = Confidence Interval

[2] False discovery rate correction for multiple testing

Supplemental Table S5: Combined results of univariate and multivariate Cox proportional hazards models for the CPTAC cohort.

CPTAC: Cox Proportional Hazard

	Univariate					Multivariate			
Characteristic	N	HR	95% CI	p-value	q-value	HR	95% CI	p-value	q-value
**AJCC Stage**	109			0.94	0.94			>0.9	>0.9
I		—	—			—	—		
II		1.14	0.54, 2.43			1.16	0.54, 2.51		
III		1.13	0.50, 2.52			1.12	0.48, 2.58		
**Age (years)**	109	0.99	0.97, 1.01	0.41	0.62	1.00	0.97, 1.02	0.7	0.8
**Hallmark Hypoxia**	109	1.27	0.98, 1.64	0.076	0.26	1.13	0.79, 1.61	0.5	0.7
**MAPK Activity Score**	109	0.91	0.69, 1.20	0.50	0.62	0.69	0.46, 1.03	0.057	0.2
**NET-Inducers**	109	1.18	0.91, 1.52	0.20	0.47	1.18	0.85, 1.64	0.3	0.7
**MPAS*NET-Inducers**	109	1.36	1.00, 1.85	0.057	0.26	1.67	1.15, 2.42	**0.008**	**0.053**
**Neutrophils (MCP-Counter)**	109	0.91	0.68, 1.22	0.53	0.62	0.87	0.63, 1.19	0.4	0.7

[1] HR = Hazard Ratio, CI = Confidence Interval

[2] False discovery rate correction for multiple testing

Supplemental Table S6: Memory T Cell Panel

Marker	Color	Clone	Company	Catalog
CD44	Alexa Fluor 488	IM7	BioLegend	103016
CD69	PE	H1.2F3	eBioscience (Thermo Fisher Scientific)	12-0691-82
Live/Dead	Live/Dead Fix Red	N/A	Thermo Fisher Scientific	L23102
KLRG1	PerCP-Cy5.5	2F1/KLRG1	BioLegend	138418
CD62L	PE-Cy7	MEL-14	BioLegend	104418
CD137	APC	1AH2	BD Biosciences	740364
CD8a	Alexa Fluor 700	53-6.7	BioLegend	100730
CD3 epsilon	Brilliant Violet 421	145-2C11	BioLegend	100341
CD27	Brilliant Violet 510	LG.3A10	BioLegend	124229
CD4	Brilliant Violet 605	GK1.5	BioLegend	100412
CD127	Brilliant Violet 650	A7R34	BioLegend	135043
CD45RA	Brilliant Violet 786	14.8	BD Biosciences	747759

Supplemental Table S7: Myeloid Cell Panel

Marker	Fluorophore	Clone	Company	Catalog
CD11b	PE-Texas Red	M1/70.15	Thermo Fisher Scientific	RM2817
CD11c	Brilliant Violet 605	N418	BioLegend	117334
CXCR2	APC	242216	Novus Biologicals	FAB2164A-100
CD206	PE	C068C2	BioLegend	141706
CD3	Brilliant Violet 785	145-2C11	BioLegend	100355
F4/80	Alexa Fluor 488	BM8	BioLegend	123120
Live/Dead Fix Aqua	Live/Dead Fix Aqua	N/A	Thermo Fisher Scientific	L34957
Ly-6C	PerCP-Cy5.5	HK1.4	eBioscience	45-5932-82
Ly-6G	V450	1A8	BD Biosciences	560603
MHC II	APC-Cy7	M5/114.15.2	BioLegend	107628

Supplemental Table S8: Stimulated T Cell Panel

Marker	Color	Clone	Company	Catalog
TIGIT	FITC	GIGD7	Thermo Fisher Scientific	11-9501-82
Lag-3	PE	C9B7W	BioLegend	125208
PD-1	PE-Cy7	RMP1-30	BioLegend	109110
TIM-3	APC	8B.2C12	Thermo Fisher Scientific	17-5871-82
CD8a	Alexa Fluor 700	53-6.7	BioLegend	100730
CD4	APC-Fire 750	RM4-5	BioLegend	100567
IFNy	Brilliant Violet 421	XMG1.2	BioLegend	505830
L/D	Live/Dead Fix Aqua	N/A	Thermo Fisher Scientific	L34957
CD137	Brilliant Violet 605	1AH2	BD Biosciences	740364
CD3 epsilon	Brilliant Violet 785	145-2C11	BioLegend	100355
IFNy Iso	Brilliant Violet 421	RTK2071	BioLegend	400429

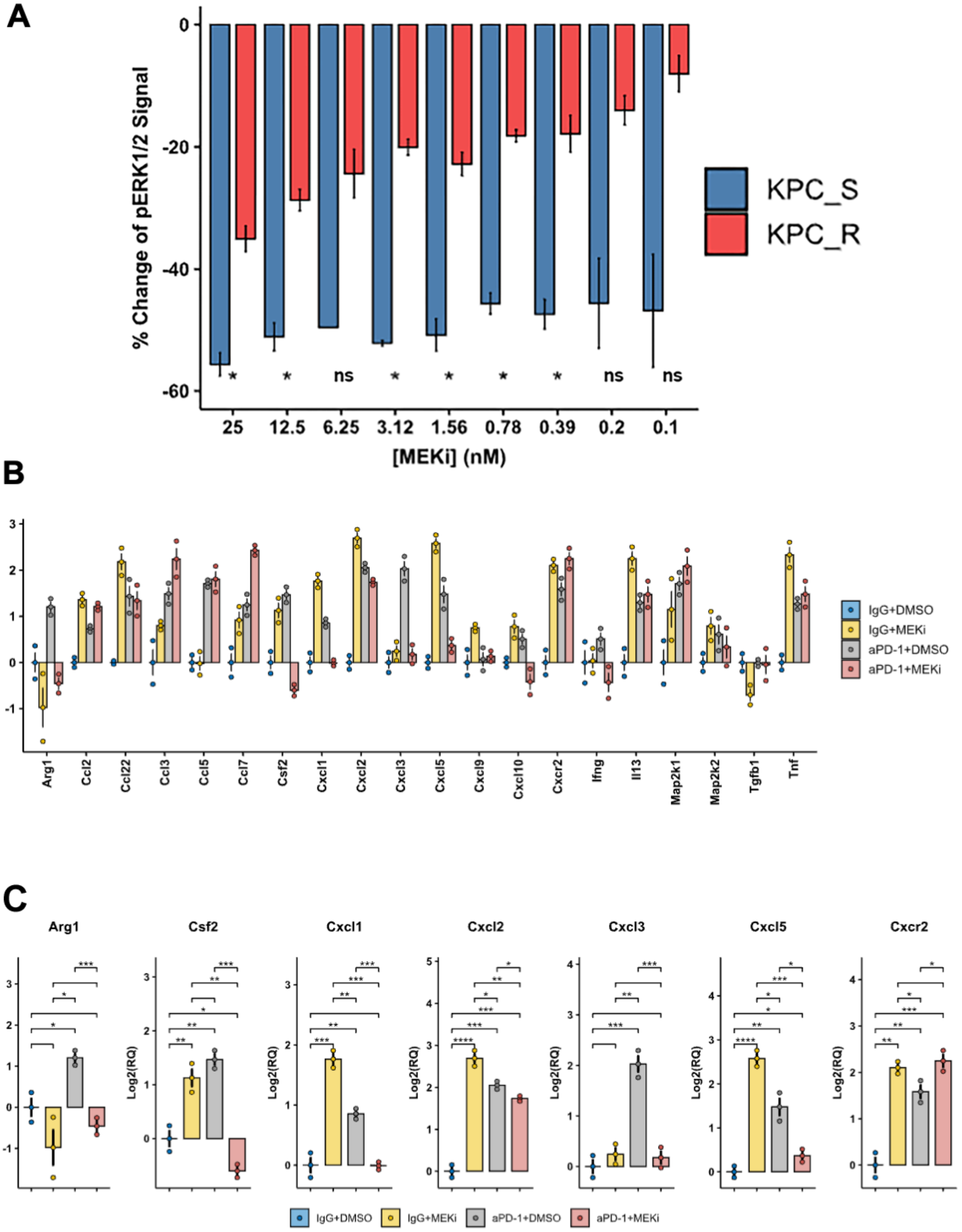

Supplemental Figure S1: Expression of pERK1/2 and neutrophil chemokines/chemokine receptors following MEKi+/-aPD-1 treatment.

A. In-cell Western blot results for KPC_S and KPC_R treated with serially diluted concentrations of Trametinib as indicated. Signal intensity of pERK1/2 was first background-corrected and then normalized to total cell count per well. Experiments were done in duplicate. **B.** RT-qPCR for a panel of selected genes. Mice were treated for 7 days followed by and pooled RNA (n=5 pooled tumors/group in triplicate runs) were assayed for relative expression against the IgG+DMSO group. **C.** Enhanced view of neutrophil-specific genes from above. Results are presented as mean +/- SEM. Statistics were performed by Kruskal-Wallis test and multiple comparisons. *$p < 0.05$, ** $p < 0.01$, *** $p < 0.001$, **** $p < 0.0001$; ns or if not shown, no significance.

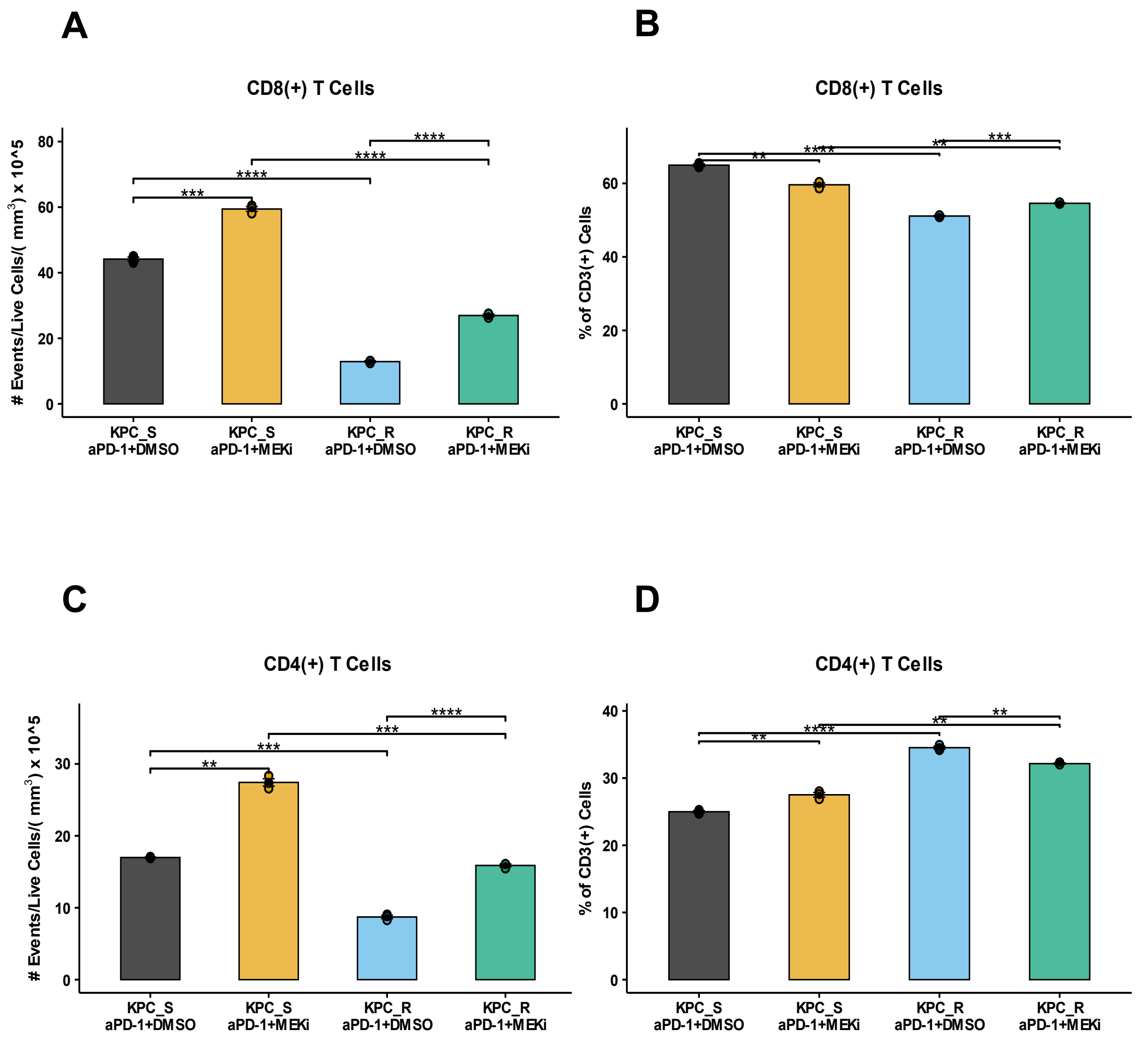

Supplemental Figure S2: aPD-1+MEKi increases CD8+ and CD4+ T cell infiltration in aPD-1+MEKi treatment-sensitive and resistant tumors.

A. Density of CD8+ T cells are increased following the aPD-1+MEKi treatment in KPC_S and KPC_R orthotopic tumors. **B.** CD8+ T cells as percent of CD3+ total T cells. **C.** Density of CD4+ T cells are increased following the aPD-1+MEKi treatment in KPC_S and KPC_R orthotopic tumors. **D.** CD4+ T cells as percent of CD3+ total T cells is increased by the aPD-1+MEKi treatment. Results are presented as mean +/- SEM of pooled replicates (n=5 pooled tumors/group in triplicate runs). Significant differences determined from Kruskal-Wallis test followed by Tukey's correction. * p< 0.05, ** p< 0.01, *** p< 0.001, **** p< 0.0001.

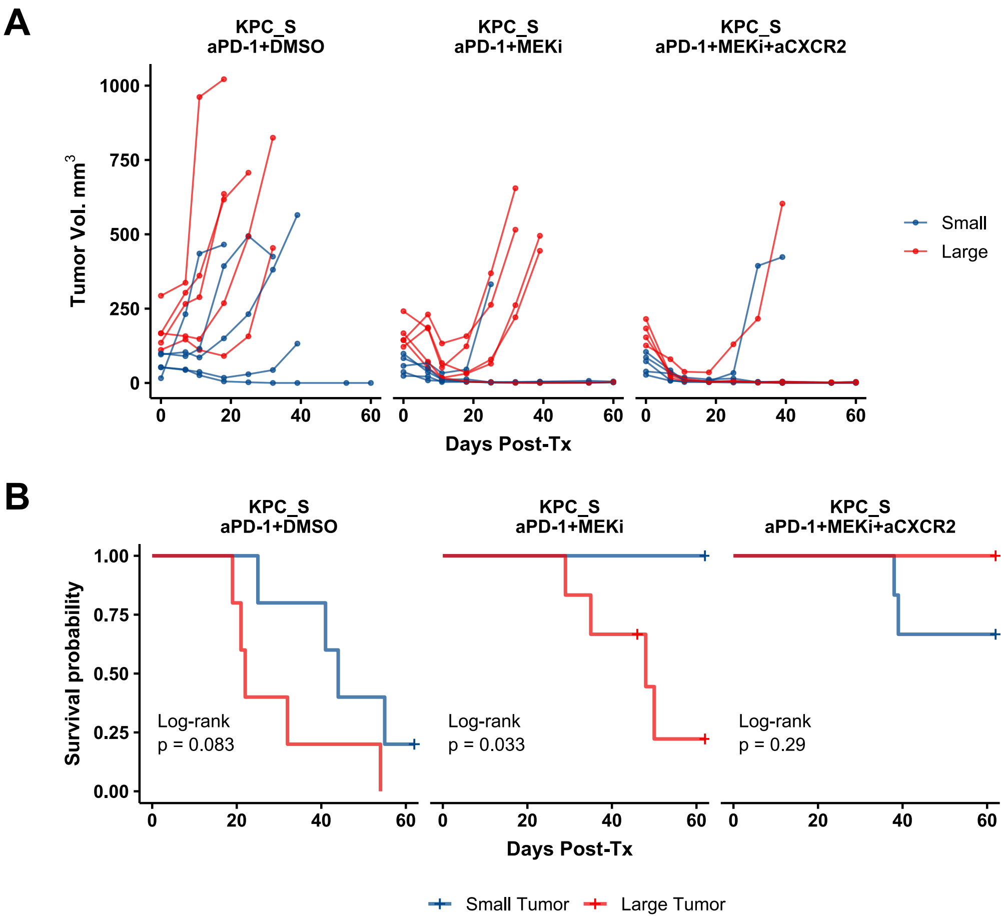

Supplemental Figure S3: Addition of aCXCR2 overcomes resistance to aPD-1+MEKi treatment in KPC_S tumors with large baseline volume.

A. Individual growth curves for KPC_S orthotopic tumors by treatment group. Ultrasound measurements of baseline tumor volume were used to categorize tumors into large or small size based on median split. **B.** Survival curves show that large baseline volume is associated with decreased survival in the aPD-1+DMSO and aPD-1+MEKi treatment groups. The triple combination with aCXCR2 results in increased overall survival. Significant differences in survival curves were determined using the Log-rank test. p values indicated.

A

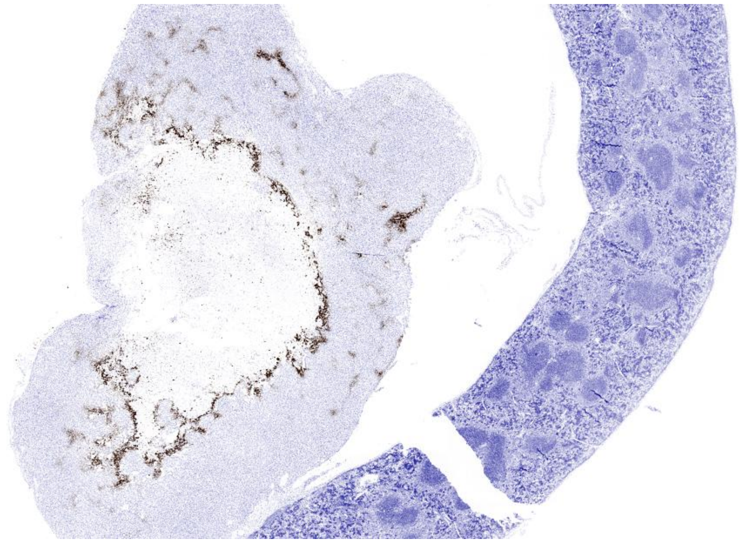

B

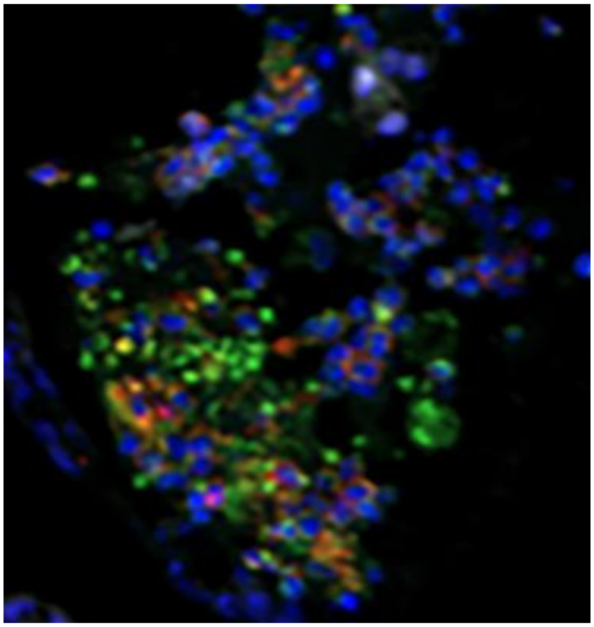

Supplemental Figure S4: Immunohistochemical representation of necrosis and immunofluorescence representation of TAN, NETosis and hypoxia markers. A. Necrosis in the KPC_S tumor**. B.** Green: MPO, Red: Ly-6G; White: hypoxyprobe.

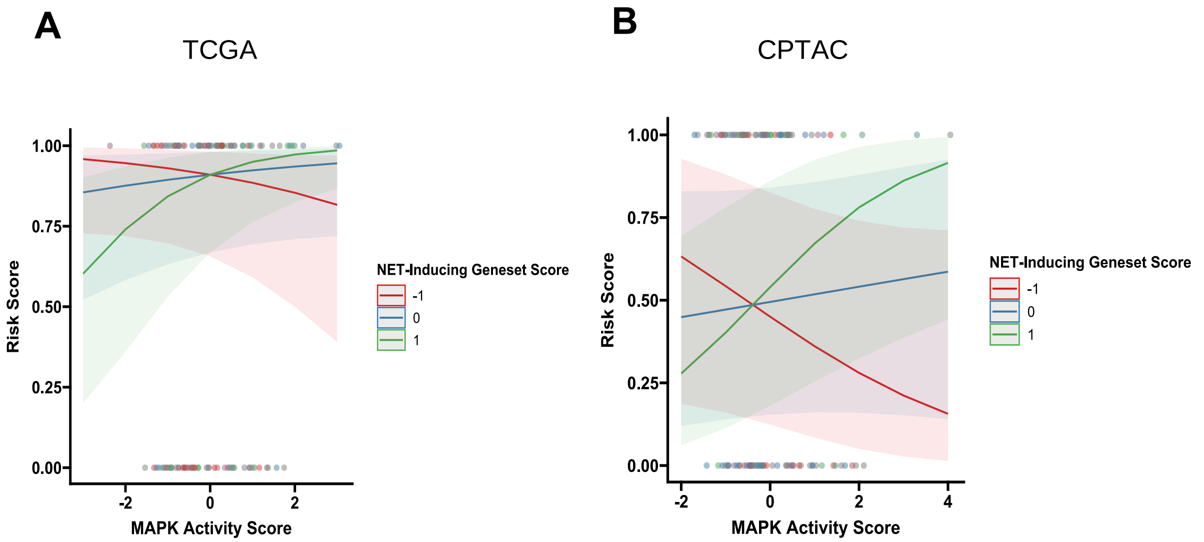

Supplemental Figure S5: Plots showing the interaction between the Overall Survival Risk Score correlations of the MAPK Activity Score and that of the NET-Inducing Gene Set Score.

A. The correlations between the MAPK Activity Score and the Overall Survival Risk Score when the NET-Inducing Gene Set Score ranges between -1 and 1 in the TCGA data set. **B.** The correlations between the MAPK Activity Score and the Overall Survival Risk Score when the NET-Inducing Gene Set Score ranges between -1 and 1 in the CPTAC data set.

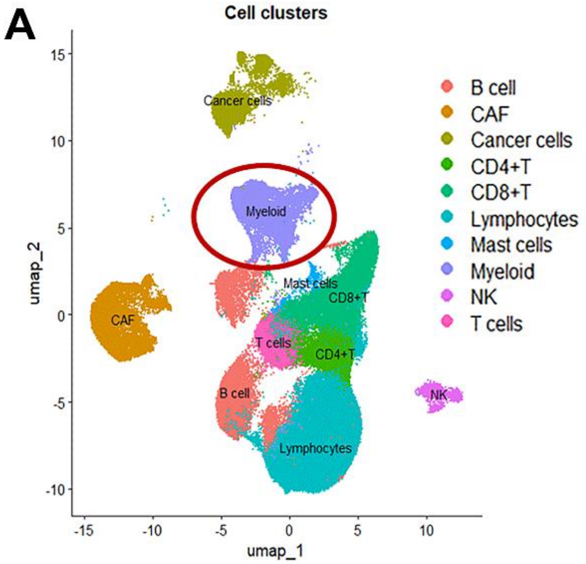

B

Cell Type	Marker Genes
B cell	MS4A1, CD19, IGHM
CD8 T cell	CD3E, CD3D, CD8A, CD8B, GZMB
CD4 T cell	CD3E, CD3D, CD4
Treg	CD3E, CD3D, CD4, FOXP3
NK cell	NCAM1, GZMB, PRF1, NKG7
Mast cells	TPSAB1
TAM	CD68, HLA-DRB1, CD163, CSF1R, CD14
DC	HLA-DRB1, LAMP3, CD80, CD86
CAF	VIM, COL12A1, COL1A2, COL6A2
Pancreatic Ductal Adenocarcinoma (PDAC, cancer)	WFDC2, VIM, EPCAM

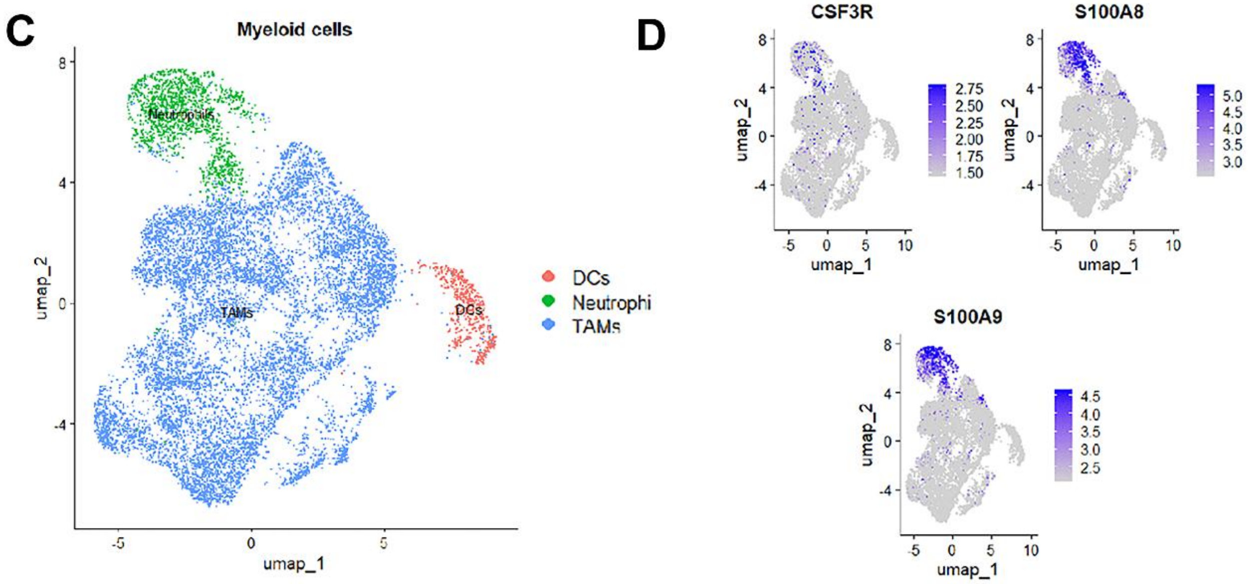

Supplemental Figure S6: Single cell analysis of resected human PDAC specimens.

A. UMAP of the major cell subtypes in PDAC. **B.** Marker genes that were used to identify the cell clusters in the UMAP. **C.** The myeloid cell cluster circled in A was further clustered into myeloid subtypes. **D.** Marker genes used for identifying neutrophils.
